# Statistical Approach for Gene Set Analysis with Trait Specific Quantitative Trait Loci

**DOI:** 10.1038/s41598-018-19736-w

**Published:** 2018-02-05

**Authors:** Samarendra Das, Anil Rai, D. C. Mishra, Shesh N. Rai

**Affiliations:** 1 Division of Statistical Genetics, ICAR-Indian Agricultural Statistics Research Institute, New Delhi, 110012 India; 2 Centre for Agricultural Bioinformatics, ICAR-Indian Agricultural Statistics Research Institute, New Delhi, 110012 India; 3 Biostatistics Shared Facility, JG Brown Cancer Center and Department of Bioinformatics and Biostatistics, University of Louisville, Louisville, 40202 KY USA

## Abstract

The analysis of gene sets is usually carried out based on gene ontology terms and known biological pathways. These approaches may not establish any formal relation between genotype and trait specific phenotype. In plant biology and breeding, analysis of gene sets with trait specific Quantitative Trait Loci (QTL) data are considered as great source for biological knowledge discovery. Therefore, we proposed an innovative statistical approach called Gene Set Analysis with QTLs (GSAQ) for interpreting gene expression data in context of gene sets with traits. The utility of GSAQ was studied on five different complex abiotic and biotic stress scenarios in rice, which yields specific trait/stress enriched gene sets. Further, the GSAQ approach was more innovative and effective in performing gene set analysis with underlying QTLs and identifying QTL candidate genes than the existing approach. The GSAQ approach also provided two potential biological relevant criteria for performance analysis of gene selection methods. Based on this proposed approach, an R package, *i.e*., GSAQ (https://cran.r-project.org/web/packages/GSAQ) has been developed. The GSAQ approach provides a valuable platform for integrating the gene expression data with genetically rich QTL data.

## Introduction

The recent advancement in genome sequencing technologies leads to generation of tremendous volume of high-throughput biological data^1^. Meanwhile, exploiting these data and drawing valid biological knowledge has posed a great challenge to scientists across the globe^2^. For instance, in genome wide expression study, the traditional objectives are (a) obtaining the expression levels of several thousand(s) of genes for the samples belonging to at least two different contrasting phenotypic/ environmental conditions, (b) identifying the genes which are relevant to these conditions under study among large pool of genes^3^. Moreover, for the later objective, several statistical and machine learning approaches have been developed^2,4^. Further, the selected genes are expected to have major causal role for the phenotypic trait under study^5^. Most of the biologists consider this as the end of their analysis. However, such analysis is the starting point of a complex process of drawing valid biological insights into high-throughput genome wide expression data^6^.

The focus in Gene Expression (GE) data analysis has been shifted from single gene to gene set level, as a gene does not work alone; rather it works as an intricate network of a set of genes^7^. Analysis of GE data in terms of gene sets has numerous computational advantageous over single gene studies^8^. Keeping in view this fact, a variety of methods for Gene Set Analysis (GSA) have been developed and used in GE analysis. The popular GSA methods include GSEA (Gene Set Enrichment Analysis)^7,9,10^, SAFE (Significance Analysis of Functional categories in gene Expression studies)^11^ and Random set methods^12^. These competitive methods compare the gene set with its complement in terms of association with previous biological knowledge base, *i.e*. pathways, Gene Ontology (GO) terms, differential expression, *etc*. under the framework of statistical hypothesis^6,13^.

Along with the development of GSA methods and expression measurement technology, the availability of other biological data like Quantitative Trait Loci (QTLs) is also growing rapidly in public domain databases. QTLs are segment of genomic regions either containing or linked to genes that correlates with variation in a phenotype (quantitative trait)^14^. Moreover, it is a classical and widely used molecular breeding method and can be a potential source for understanding the genotype-phenotype relationships in plant biology. Further, the causal relation between variation in a specific trait and differences in underlying genotypic level is of paramount importance for understanding genome function and evolution^15^, which is the basis for targeted molecular breeding.

Therefore, performing analysis of gene sets based on trait specific QTLs through a computational approach instead of traditional GO or pathways information will be very helpful in unraveling genotype-phenotype relationships. The enrichment analysis of gene sets is well developed in human disease genetics, where, GO terms and known biological pathways are taken into account^16–18^. These approaches may not be useful to establish any formal relation between genotype and trait specific phenotype in plants. Thus, in plant biology and breeding, analysis of gene sets with trait specific QTLs requires innovative and advanced statistical techniques.

In this study, we propose an innovative statistical approach for analysis of gene sets with trait specific QTLs (GSAQ) under a sound computing framework. Further, its utility was evaluated on five complex abiotic and biotic stresses in rice (*Oryza sativa* L.), as rice genome is well annotated. The performance analysis of the GSAQ approach indicated its effectiveness and efficiency in performing the trait specific enrichment analysis of gene sets through incorporating background QTL information. This proposed technique also able to integrate GE data with QTL data to provide effective gene sets enriched with the QTL information. The developed statistical approach was embodied in the form of an R software package for the users. Further, we also illustrated the application of the developed GSAQ approach as biological relevant criteria to evaluate the performance of gene selection methods based on high dimensional GE data. For this purpose, we used ten different gene selection methods, *viz*. Support Vector Machine-Recursive Feature Elimination (SVM-RFE), t-score, F-score, Maximum Relevance Minimum Redundancy (MRMR) technique, Random Forest (RF), Information Gain (IG), Gain Ratio (GR), Symmetrical Uncertainty (SU), Pearson’s Correlation Filter (PCF) and Spearman’s Rank Correlation (SRC)^19–28^. Our results showed that, GSAQ approach provided two biological relevant criteria for evaluating the performance of gene selection methods on GE data.

## Materials and Methods

The performance analysis of the proposed GSAQ approach was carried out on rice, as it is a model crop plant and huge amount of GE and QTL datasets are publicly available. Therefore, five different GE datasets related to two biotic stresses *i.e*., blast (fungal) and brown plant hopper (insect) and three abiotic stresses *i.e*., salinity, cold and drought for rice were collected. These GE datasets were obtained from Gene Expression Omnibus (GEO) database of NCBI (http://www.ncbi.nlm.nih.gov) with platform GPL2025, as this platform contains 191 GE experiments (series) comprising 3096 samples/subjects of rice. Among these samples, 304 experimental samples related to these biotic and abiotic stresses for rice were taken in this study through performing meta-analysis individually for each of the stresses. The detail process of meta-analysis is given in Supplementary Document S1. The summary and detail descriptions of the GE datasets are given in Supplementary Table S1 and S2 respectively. Further, the trait specific QTLs for the stresses, *viz*. fungal, insect, salinity, drought and cold (for which the GE datasets were obtained from GEO database of NCBI) for rice were collected from the Gramene QTL database (http://www.gramene.org/qtl)^29^.

### Data preprocessing

The preprocessing of the data was intended to remove noises, including missing probes and mislabeled probes^5^. For this purpose, the analysis was conducted by using Bioconductor platform of R. Initially, the raw CEL files of the collected samples were processed using Robust Multichip Average (RMA) algorithm available in *affy* Bioconductor package of R^30,31^. This includes background correction, quantile normalization and summarization by median polish approach^32^. Further, the log2 scale transformed expression data from RMA for the selected experimental samples were used for further selection of relevant gene sets.

### Preliminary gene selection for dimension reduction

For tens of thousands of genes in GE data, it would be of high computational complexity to use the gene set selection methods directly^3^. Hence, we first employed t-test and Fold Change (FC) criteria to filter out unlikely genes to reduce the dimension of the GE datasets. In our preliminary selection, we assigned 1 and 0.05 as the |FC| and *p-value* thresholds respectively, resulting in selection of several thousands of genes (Supplementary Table S1). The detail procedure of preliminary gene selection is given in Supplementary Document S2. Further, GE data on these selected genes (at the preliminary stage) were further used for final gene set selection using different gene set selection methods.

### Selection of gene sets

Among the thousand(s) of genes in GE datasets, it is challenging from systems biology point of view to choose the set of genes that are most relevant to the specific trait^4^. Here, we have taken eight statistical methods, *viz*. t-score, F-score, MRMR, IG, GR, SU, PCF, SRC and two machine-learning methods, *viz*. RF and SVM-RFE to select relevant gene sets (Supplementary Table S3). These ten gene selection methods were applied on high dimensional GE datasets related to five different stresses for selection of pertinent gene sets of rice. For all gene selection methods, the gene lists were prepared by arranging the genes based on the descending order of the respective computed metrics. The gene sets of different sizes were selected from the prepared gene lists through each gene selection method for each stress. Detail procedure for selection of relevant gene sets from high dimensional GE datasets by each of these 10 methods is given in Supplementary Document S2.

### Proposed approach for Gene Set Analysis with QTL (GSAQ)

Let **Ω** be the whole gene space (set of genes in a genome), *G* be a selected gene set obtained by using a gene selection method for a particular condition/ trait, *G*′(*i.e*. **Ω**–*G*) be the set of not selected genes *i.e*. complement of *G*. Let, *N* and *n* be the number of elements in **Ω** and *G* respectively. Let *Q* be the set of associated QTLs for the same trait. Suppose, for a member gene (*i-th* gene) in *G*, *i.e. g*_*i*_^c^ [*a*, *b*] ϵ *G*, where *a* and *b* represent start and stop positions (in terms of base pairs) of the gene *g*_*i*_ in chromosome *c*. Similarly, for a member QTL (*t-th* QTL) in *Q*, *i.e. q*_*t*_^c^[*d*, *e*] ϵ *Q*, where, *d* and *e* represent the start and stop positions of the QTL *q*_*t*_ on chromosome *c*. The complete overlap of the genomic regions of the gene *g*_*i*_^*c*^ with that of a QTL *q*_*t*_^c^ can be expressed by using an indicator function, which is shown as:

1 $${I}_{{q}_{t}}({g}_{i})=\{\begin{array}{ll}1 & if\,{g}_{i}^{c}[a,b]\in {q}_{t}^{c}[d,e]\\ 0 & if\,{g}_{i}^{c}[a,b]\notin {q}_{t}^{c}[d,e]\end{array}$$

In other words, the selected gene will have a QTL hit, if its genomic regions completely overlapped with that of a QTL for a particular trait (both belong to the same chromosome). Further, the total number of genes in *G* overlapped with QTL regions can be defined by a statistic called as total number of QTL hits (*NQhits*) in *G* and given as:

2 $$NQhits=\sum _{t=1}^{|Q|}\sum _{i=1}^{n}{I}_{{q}_{t}}(\,{g}_{i})$$

Besides this, the proportions of genes those got QTL hits (*Pr*_*GQ*_) in *G* can also be computed by:

3 $$P{r}_{GQ}=\frac{NQhits}{n}$$

Similarly, proportions of genes those got QTL hits in *G*′($P{r}_{G^{\prime} Q}$) can be expressed as:

4 $$P{r}_{G^{\prime} Q}=\frac{NQhits^{\prime} }{N-n}$$

where, *NQhits*′ be the total number of QTL hits in *G*′.

The expressions in Eqs 1 and 2 can be used to show whether a gene got a QTL hit or not and to compute the *NQhits* statistic for all genes in *G* respectively. The developed statistic may not be sufficient to evaluate the statistical significance of selected gene set related with the specific trait. To this end, Wang *et al*.^5^ proposed the Gene Set Validation with QTLs (GSVQ) (or Microarray-QTL) test using Hypergeometric distribution to validate the selected salinity responsive genes in rice with salinity QTLs^5^. However, GSVQ test is unique of its kind, but it is not statistically sound as it violates the basic assumptions of Hypergeometric distribution (*i.e*. sampling without replacement) and fails to state the underlying null hypothesis.

Therefore, to perform the gene set analysis with the underlying trait specific QTLs under a sound computing framework, we developed the GSAQ approach. In other words, it can be used to evaluate the statistical significance of selected gene sets related to specific trait based on available QTL information. Under this approach, the following hypothesises can be constructed for testing purpose.

*H*_0_: Genes in *G* are at most as often overlapped with the QTL regions as the genes in *G*′(*i.e*. $P{r}_{GQ}=P{r}_{G^{\prime} Q}$)

*H*_1_: Genes in *G* are more often overlapped with the QTL regions as compared to genes in *G*′ (*i.e*. $P{r}_{GQ} > P{r}_{G^{\prime} Q}$)

In other words, the above constructed null hypothesis is a competitive one as it considers the genes from both *G* and *G*′^6^.

The proposed GSAQ approach is based on formation of 2 × 2 contingency tables and Hypergeometric distribution. Further, the 2 × 2 tables were extensively used in differential expression analysis, GO and pathways enrichment analysis^6,11,33^. The basic concept behind this 2 × 2 table method is a gene sampling model. Moreover, each cell of such table is filled with a sample of genes, each of which is drawn at random from the gene space. Here, in this sampling model, each sampling unit (*i.e*. gene) can be subjected to two fixed set of indicator measurements, *i.e*. (*A*, *B*), where, (i) *A* (1 or 0) indicates whether the gene is a part of the selected gene set or not and (ii) *B* (1 or 0) indicates whether that gene got the QTL hit or not. Further, the gene space can be formalized into a population having *N* units (for *N* genes) and shown as:

5 $$({A}_{1},{B}_{1}),({A}_{2},{B}_{2}),\ldots ,({A}_{i},{B}_{i}),\ldots ,({A}_{N},{B}_{N})$$

where, *i-th* unit *i.e*. (*A*_*i*_, *B*_*i*_) shows that whether *i-th* gene is a part of the gene set or not and whether it also got QTL hit or not.

Here, the gene sampling model (where genes are taken as sampling units) is quite different from the usual classical subject sampling model (where the GE profiles are considered as sampling units)^6^. Through this gene sampling model (by fixing A_*i*_ = 1), *K* gene samples, *i.e. G*_1_, *G*_2_, …, *G*_*K*_, each of size *m* (≤*n*) are randomly drawn from the population with equal probability by using simple random sampling without replacement procedure. For each *G*_*k*_ (*k* = 1, 2, …, *K*), a 2 × 2 table, as shown in Table 1, was constructed. Similarly, using this procedure, *K*, 2 × 2 contingency tables were obtained for *K* gene samples. The *NQhits* statistic computed through Eq. 2 from 2 × 2 table (Table 1) constructed for *k-th* gene sample follows a Hypergeometric distribution^5^ and given as:

6 $$P[X={N}_{{G}_{k}Q}]=\frac{(\begin{array}{c}{N}_{Q}\\ {N}_{{G}_{k}Q}\end{array})(\begin{array}{c}N-{N}_{Q}\\ m-{N}_{{G}_{k}Q}\end{array})}{(\begin{array}{c}N\\ m\end{array})}$$

where, *X* is a random variable representing the value of *NQhits* (*N*${G}_{k}Q$) for *k-th* gene sample (*k* = 1, 2, …, *K*), *N*_*Q*_ is total number of QTL hits in Ω and *m* is the size of *k-th* gene sample.

**Table 1:** 2 × 2 contingency table for gene set enrichment test with QTL.

	Overlapped with QTL regions	Not overlapped with QTL regions	Total
Selected gene set	*N*_*Gk*__*Q*_	*N*_*Gk*__*Q*_^*c*^	*N*_*Gk*_
Not selected gene set	*N*_*Gk*_^*c*^_*Q*_	*N*_*Gk*_^*c*^_*Q*_^*c*^	*N*_*Gk*_^*c*^
Total	*N*_*Q*_	*N*_*Q*_^*c*^	*N*

**Ω**: gene space; *N*_*Gk*_: number of genes in _*Gk*_; *N*_*Gk*_^*c*^: number of genes in _*Gk*_^*c*^; *N*_*Q*_: number of QTL hit genes in gene space: *N*_*Q*_^*c*^: number of non-QTL hit genes in gene space. _*Gk*_: *k*-th gene sample from selected gene set; _*Gk*_^c^: **Ω**-_*Gk*_.

Through the Hypergeometric distribution (Eq. 6), the statistical significance value or *p-value* (*p*_*k*_) for *k-th* gene sample was computed by using Eq. 7.

7 $${p}_{k}=P[{X}_{k}\ge x|{H}_{0}]=1-P[{X}_{k}\le x|{H}_{0}]$$

For assessing the final statistical significance of the test, the individual *p-values* needs to be combined.

### Methods for combining p-values

Suppose, there are *K* independent tests (for *K* random gene samples) and their associated *p-values* are *p*_1_, *p*_2_, …, *p*_*K*_. Under *H*_0_, the *p-values* from individual gene samples are uniformly distributed between 0 and 1 (*i.e.p*_*k*_ ~ U [0, 1])^34^. To get the overall statistical significance value for the test (*H*_0_ *vs. H*_1_), the individual *p-values* for each gene samples can be combined. For this purpose, the methods described in Table 2 can be used.

**Table 2:** List of methods used for combining *p-values* to assess final enrichment significance.

Methods	Transformed variable	Test statistic	Dist. under *H*_0_	Reference
Inverse Normal	*Z*_*k*_ = Φ^−1^(*p*_*k*_)	$T={\sum }_{k=1}^{K}{w}_{k}{Z}_{k}$	N (0, 1)	^39^
Meanp	$\bar{p}={\sum }_{k=1}^{K}{p}_{k}/K$	$W=(0.5-\bar{p})\sqrt{12\,K}$	N (0, 1)	^40^
Inverse Chi-Square	*Z*_*k*_ = −2log*p*_*k*_	$L={\sum }_{k=1}^{K}{Z}_{k}$	χ²_2Kdf_	^41,42^
Logit	*S*_*k*_ = log[*p*_*k*_/(1−*p*_*k*_)]	$S={\sum }_{k=1}^{K}{S}_{k}$	t_5K+4 df_	^43^

*p*_*k*_: statistical significance value of *k-th* gene sample; *Φ*, *Φ*^−1^: standard normal cumulative distribution function and its inverse respectively; *K*: Number of random gene samples; df: degrees of freedom; *H*_0_: Competitive null hypothesis; *N*(): Normal distribution; t: Central t-distribution; χ²: Central Chi-square-distribution.

**Figure 1:**
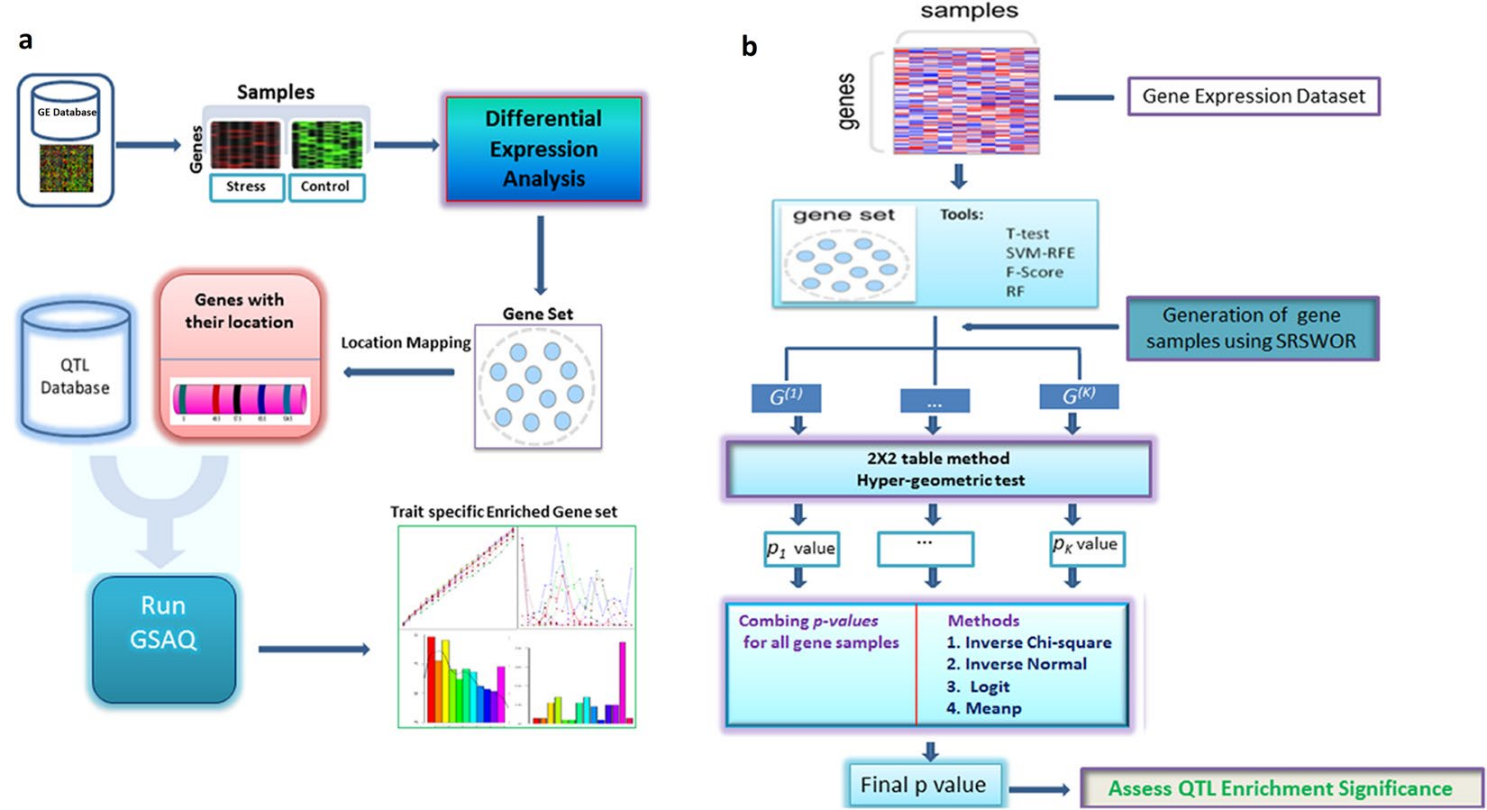
Operational procedure and algorithm of GSAQ approach. (**a**) Operational procedures involved in GSAQ are shown in pictorial form. GE reperesents Gene Expression (**b**) Flowchart of the computational algorithm implemented in GSAQ approach. G^(*k*)^’s represents random gene samples and *p*_*k*_-values represent corresponding statistical significance for each G^(*k*)^. SRSWOR represents simple random sampling without replacement.

Using the above approach, the final statistical significance values (*p*-*values*) and False Discovery Rate (FDR) values for the selected gene sets were computed. In this case, the gene sets were selected using ten existing gene selected methods as given in Supplementary Table S3. For the computation of *p-values*, we took different combinations of *m* and *K* for selected gene sets as given in Supplementary Table S4. The performance analysis of the proposed GSAQ approach and gene selection methods was carried out on complex abiotic and biotic stresses, *viz*. salinity, cold, drought, fungal and insect, in rice. Moreover, for the computation of FDR for each selected gene set, we executed the *fdrtool* function implemented in *fdrtool* R package^35^ which is based on the approach developed by Strimmer (2008)^36^. The operational procedure of the GSAQ approach and its implemented algorithm are shown in Fig. 1. The inputs for GSAQ tool is briefly described in Supplementary Document S4.

### Developed GSAQ R software package

To facilitate the use of the proposed approach, we have developed resources that are freely available from the CRAN site of R. This resource includes the GSAQ R package, accompanying documentation and model real data examples. This package can be freely downloaded from https://cran.r-project.org/web/packages/GSAQ. This software is capable of computing *NQhits* statistic and performing QTL specific gene set enrichment analysis through the proposed GSAQ and existing GSVQ approaches. Besides, it can also be used for selection of relevant gene sets from high-dimensional GE data through different gene selection methods, obtaining QTL candidate genes and getting chromosome and QTL wise distributions of genes in selected gene set.

## Results

### Selection of gene sets

Using high dimensional GE datasets pertain to various biotic and abiotic stresses, we selected different gene sets of sizes, *viz*. 100, 200, 300, …, 2000 through a two-stage approach of preliminary gene selection and ten different gene selection methods, which are relevant to individual traits/stresses in rice. Further, we mapped the QTLs and genes in each selected gene set (for each gene selection methods) in the whole genome using MSU rice genome browser^37^. The list of QTLs detected for the stresses, *viz*. salinity, cold, drought, fungal and insect, along with their genomic regions are given in Supplementary Document S3.

### Distribution of NQhits statistic

**Figure 2:**
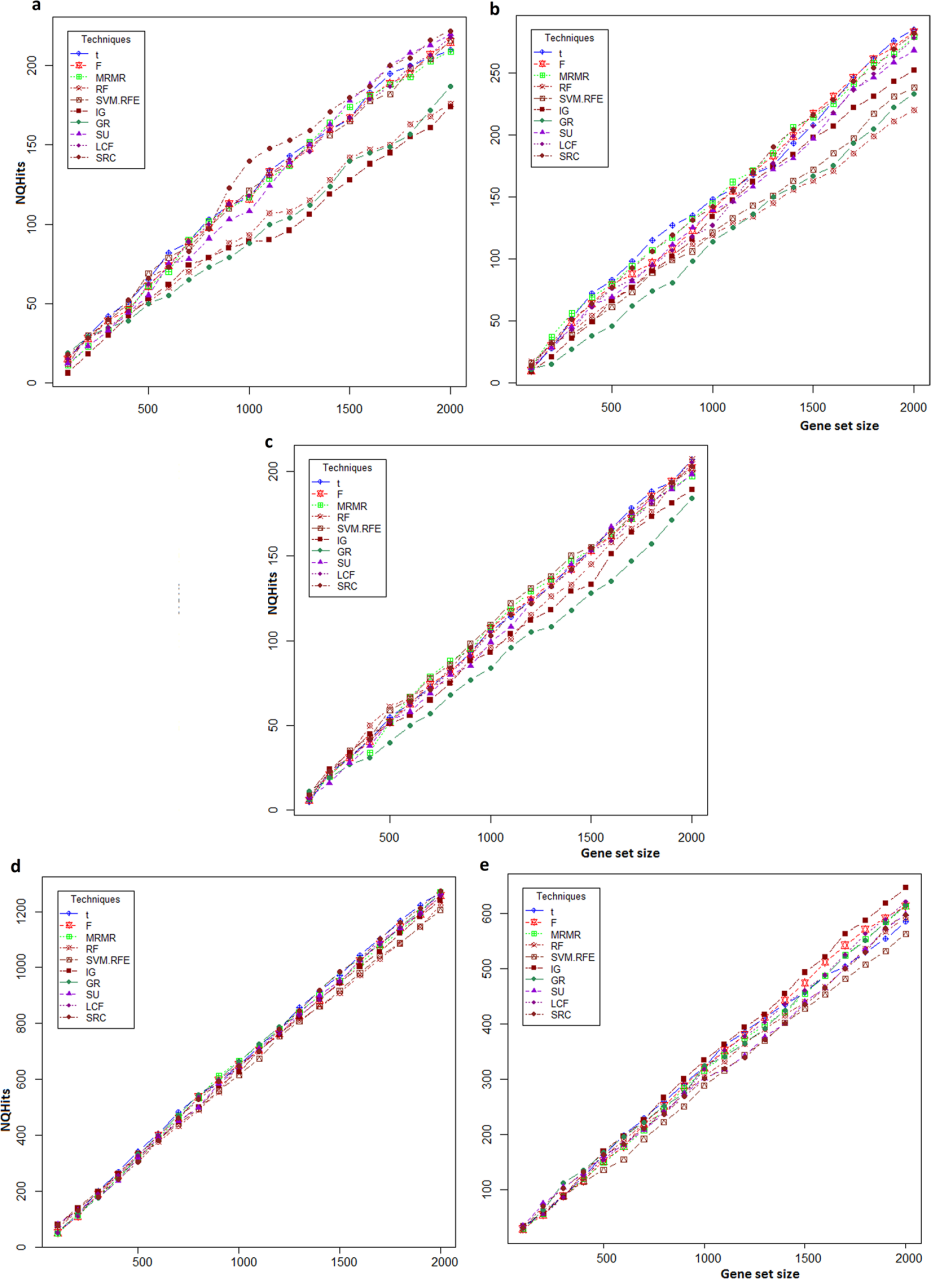
Distribution of *NQhits* statistic over the selected gene sets. The horizontal axis represents the gene sets obtained by each of the ten gene selection methods. The vertical axis represents *NQhits* statistic obtained through GSAQ approach. Distribution of *NQhits* are shown for (**a**) salinity, (**b**) cold, (**c**) drought, (**d**) fungal and, (**e**) insect stresses in rice.

The distributions of *NQhits* statistic over gene sets obtained by ten different gene selection methods for each of these five stresses are shown in Fig. 2. For all these stresses, the value of *NQhits* statistic is found to be directly proportional to size of the gene set (Fig. 2). In other words, the value of *NQhits* statistic depends on the factors like length of QTLs and number of genes in a gene set linked to QTLs for a given stress. This observation is true for all gene selection methods irrespective of stresses. Moreover, the developed *NQhits* statistic can also be used as a metric for evaluating the performance of gene selection methods. The performance of different gene selection methods based on *NQhits* statistic for the abiotic stresses, *viz*. salinity, cold and drought are at par for selection of smaller gene sets, as the value of *NQhits* for relative smaller gene set sizes (*e.g*. 100–500) are almost equal (Fig. 2). But, in case of larger gene sets, the performance based on *NQhits* statistic is found to be better for t-score, F-score, MRMR, SU, PCF, SRC and SVM-RFE as compared to IG, GR and RF. On the contrary, for the biotic stresses (fungal and insect) most of the gene selection methods performed equally well over various gene sets in terms of *NQhits* statistic (Fig. 2). This variation in performance of gene selection methods under abiotic stresses may be due to the complex/polygenic nature of abiotic stresses (due to non-living climatic factors) as compared to biotic stresses (living factors).

### Gene sets analysis with QTLs

Although the *NQhits* statistic can be used as a performance evaluation metric but, it failed to tell the trait specific enrichment of gene sets or association of genotype-phenotype relation. Therefore, we proposed GSAQ approach to test the trait specific enrichments of the gene sets with underlying QTLs. For this purpose, gene sets were selected from the high dimensional GE data by using ten different methods (Supplementary Table S3). Further, we explored the ability of the proposed GSAQ approach along with existing GSVQ approach to provide biologically meaningful insights (*e.g*. establishing genotype-trait specific phenotype associations) in five complex abiotic and biotic stresses in rice. For both the approaches, we searched significantly associated gene sets enriched with underlying QTLs, which were selected by a particular gene selection method in each of the stresses.

**Figure 3:**
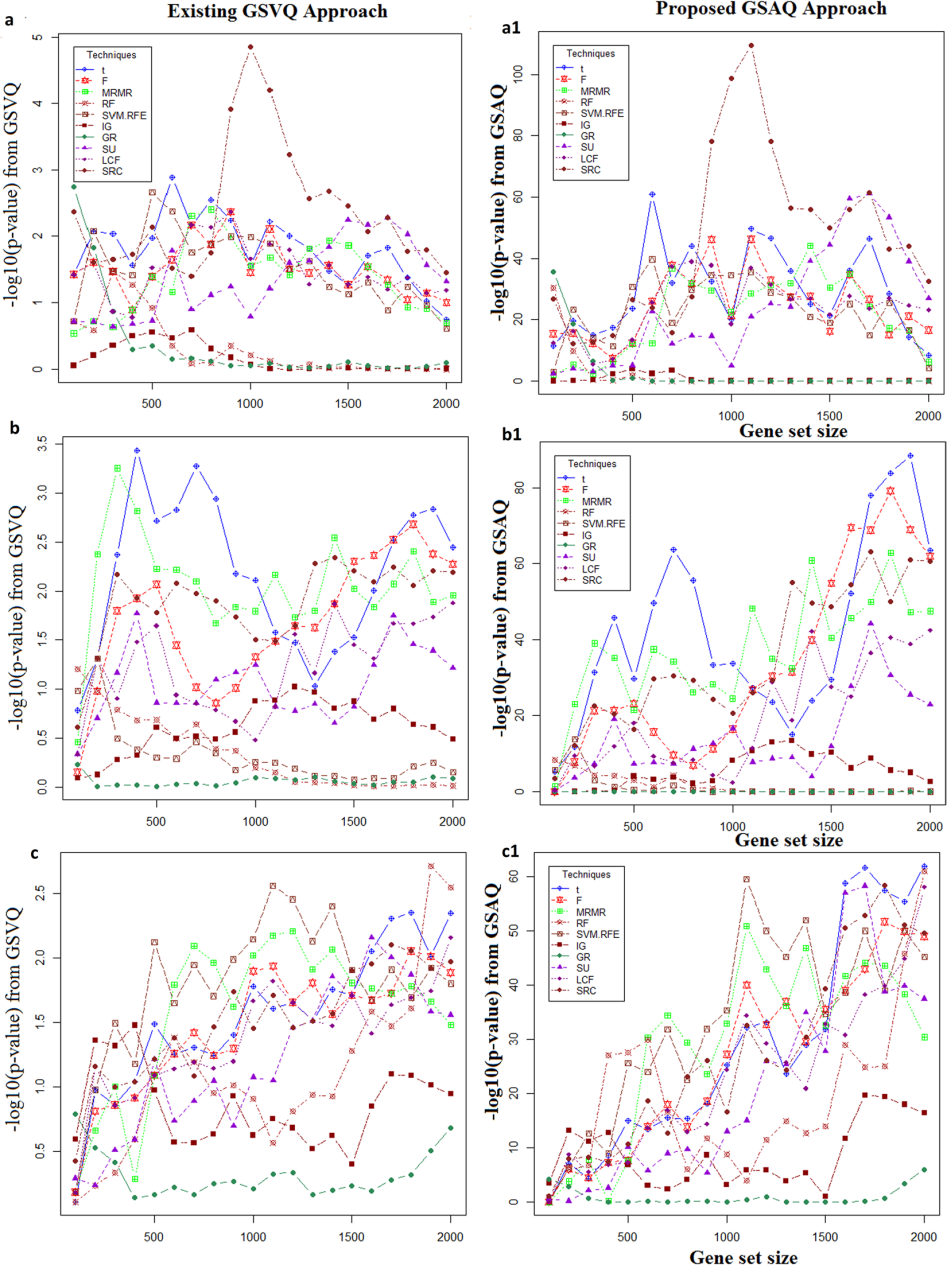
Performance analysis of GSAQ approach with GSVQ for abiotic stresses. The horizontal axis represents the gene sets obtained by each of the ten gene selection methods. The vertical axis shows the *negative logarithm of statistical significance values* computed from existing GSVQ approach for (**a**) salinity, (**b**) drought, (**c**) cold stresses and proposed GSAQ approach (with Inverse normal method) for (**a1**) salinity, (**b1**) drought, (**c1**) cold stresses.

**Figure 4:**
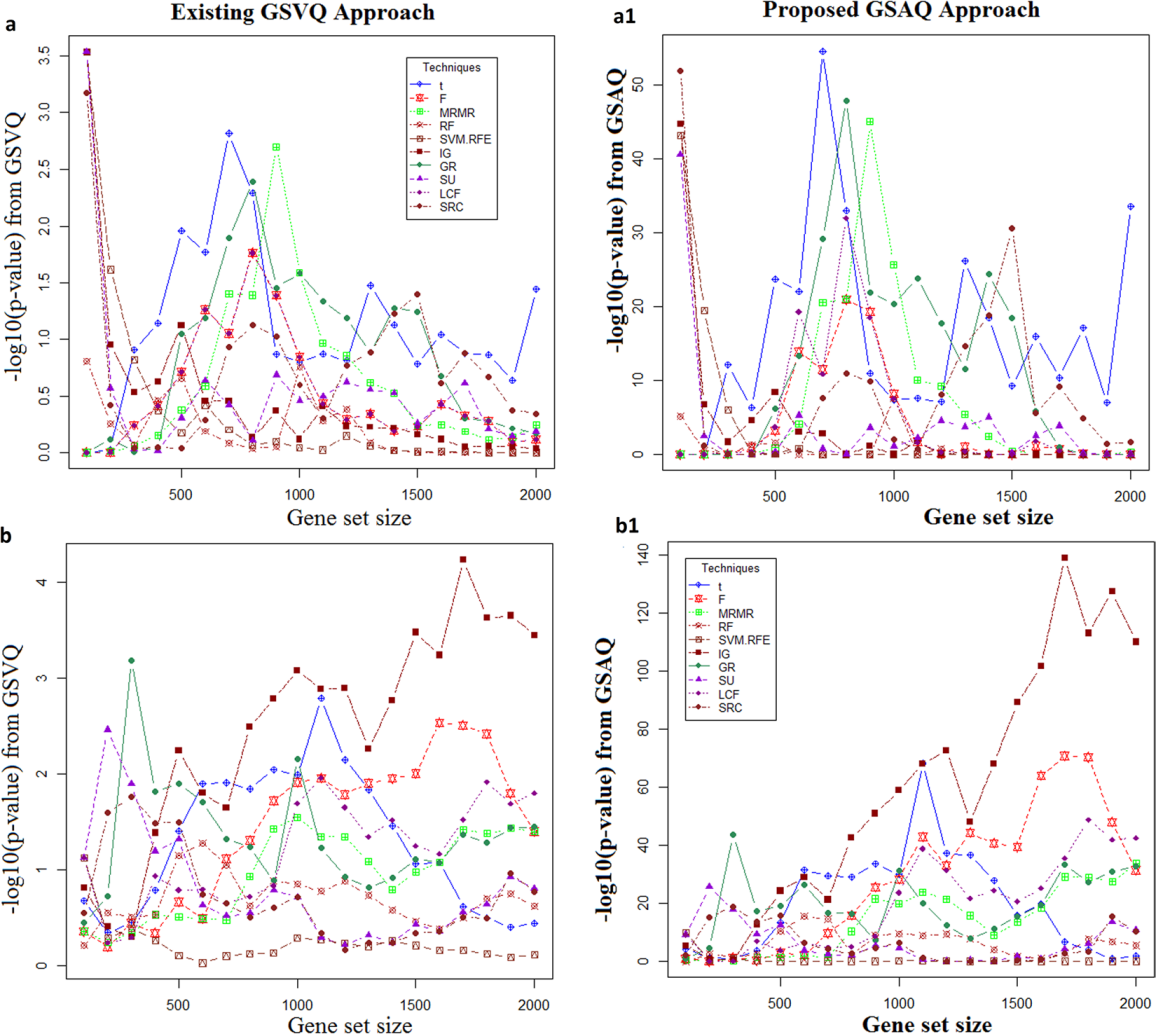
Performance analysis of GSAQ approach with GSVQ for biotic stresses. The horizontal axis represents the gene sets obtained by each of the ten gene selection methods. The vertical axis shows the *negative logarithm of statistical significance values* computed from existing GSVQ approach for (**a**) fungal, (**b**) insect stresses and proposed GSAQ approach (with Inverse normal method) for (**a1**) fungal, (**b1**) insect stresses in rice.

The distribution of *p-values* computed from both existing GSVQ and proposed GSAQ approaches are shown in Figs 3, 4 and S2–S4. For salinity stress, the distribution of *p-values* computed from GSAQ using Inverse normal method for all gene sets (through all gene selection methods) are shown in Fig. 3a1. It has been observed that except IG, GR and RF, all gene selection methods provided gene sets which were highly statistically significant at 0.001% level of significance (as *p-values* < 10E-5) (Fig. 3a1). These findings clearly indicate that the gene sets obtained by most of the methods are enriched with underlying trait specific QTLs through our GSAQ approach. Similar interpretations can be made for all other methods as given in Table 2 for GSAQ test for same stress considered in this study (Supplementary Figures S2–S4).

On the contrary, when existing approach GSVQ was used for performing salinity trait specific enrichment analysis, none of the gene sets selected by any method (except gene sets of sizes 900–1200 from SRC) are found to be significant at the same level of significance (Fig. 3a). Such findings may not be valid as per our expectation as these gene sets are selected by most powerful contemporary gene selection methods *like* SVM-RFE, RF, GR, SU, t-score, *etc*.

Further, the magnitude of −log10 (*p-values*) from GSAQ enrichment analysis for salinity stress (Fig. 3a1) is found to be much higher than that of existing GSVQ test (Fig. 3a). In other words, GSAQ approach more often rejects *H*_0_ (*i.e*. equal salinity QTL enrichment of both selected and not selected gene sets) as compared to GSVQ test. Therefore, it is found that salinity trait specific gene set enrichment analysis was better through GSAQ as compared to GSVQ. In order to cross validate these findings on the same datasets related to salinity stress, we computed FDR for both GSAQ and GSVQ for all gene sets. The results are given in Table 3 and S5. It has been observed that the value of FDR from proposed GSAQ approach for all these gene sets irrespective of gene selection methods are far below than that of existing GSVQ test (Table 3 and S5). Therefore, it can be concluded that the proposed GSAQ is more efficient than the GSVQ for performing gene set enrichment testing with salinity trait specific QTLs.

**Table 3:** Performance analysis of GSAQ and GSVQ approaches for salinity stress in rice.

Methods	100	200	300	400	500	1000	2000
t	<0.5	<0.01	<0.001	<0.001	<0.001	<0.0001	<0.0001
	(>0.5)	(>0.01)	(>0.01)	(>0.01)	(>0.01)	(>0.1)	(>0.01)
F	<0.5	<0.01	<0.001	<0.001	<0.001	<0.0001	<0.0001
	(>0.5)	(>0.01)	(>0.01)	(>0.01)	(>0.01)	(>0.01)	(>0.01)
MRMR	<0.01	<0.01	<0.01	<0.01	<0.0001	<0.0001	<0.0001
	(>0.1)	(>0.1)	(>0.01)	(>0.01)	(>0.01)	(>0.1)	(>0.01)
SU	<0.1	<0.1	<0.0001	<0.0001	<0.0001	<0.0001	<0.0001
	(>0.1)	(>0.1)	(>0.1)	(>0.2)	(>0.01)	(>0.01)	(>0.5)
PCF	<0.01	<0.01	<0.01	<0.01	<0.01	<0.0001	<0.0001
	(>0.01)	(>0.01)	(>0.01)	(>0.01)	(>0.01)	(>0.5)	(>0.5)
SRC	<0.01	<0.01	<0.01	<0.01	<0.01	<0.0001	<0.0001
	(>0. 01)	(>0.1)	(>0.01)	(>0.01)	(>0.01)	(>0.001)	(>0.001)
SVM	<0.01	<0.01	<0.01	<0.01	<0.0001	<0.0001	<0.01
	(>0.1)	(>0.01)	(>0.01)	(>0.01)	(>0.1)	(>0.1)	(>0.1)

FDR: False discovery rate; Gene sets: gene sets obtained from each method; (.): the values in parentheses indicate the FDR value computed through GSVQ approach; t: t-score; F: F-score; MRMR: Maximum Relevance Minimum Redundancy; SU: Symmetrical Uncertainty; PCF: Pearson’s Correlation Filter; SRC: Spearman’s Rank Correlation filter; SVM: Support Vector Machine with Recursive Feature Elimination.

For drought and cold stresses, none of the gene sets selected by any of the ten gene selection methods considered in this study, are found to be enriched with the respective stress specific QTLs, when enrichment analysis was performed through GSVQ approach (Fig. 3b and c). However, all selected gene sets (for drought and cold stresses), irrespective of the gene selection methods (except GR) are found to be more enriched with underlying QTLs through the proposed GSAQ approach using Inverse normal method (Fig. 3b1 and c1). Further, the −log10 (*p-values*) computed through GSAQ approach (Fig. 3b1 and c1) are found to be much higher than that of GSVQ test for drought and cold stresses (Fig. 3b and c). Subsequently, it was also verified that the FDR value for all gene sets from the GSAQ approach is found to be lesser than that of GSVQ for these stresses (Supplementary Table S5). Similar results are also obtained for other methods used in GSAQ approach (Supplementary Figures S2–S4).

Therefore, it can also be concluded that like salinity stress, the proposed GSAQ approach is found to be better and more efficient than GSVQ for performing QTLs specific gene set enrichment testing for drought and cold stress. Further, similar interpretations for the GSVQ and GSAQ approaches can be made for the biotic stresses (insect and fungal) (Figs 4 and S2–S4). Therefore, it has been observed that the GSAQ approach performs QTL enrichment analysis of gene sets more successfully and efficiently as compared to existing GSVQ test, when there is sufficient background QTL information available.

Our analysis showed that we find much greater consistency in QTL specific gene set enrichment analysis across five different stress scenarios, *viz*. salinity, cold, drought, fungal and insect, by using GSAQ than GSVQ (Figs 3 and 4).

### Performance analysis of gene set selection methods based on proposed GSAQ

Apart from assessing the significance of the genotype (gene set) to phenotype (trait) enrichment test, GSAQ can also be used as a performance evaluation metric of gene selection methods for high dimensional GE data. For instance, in salinity stress, 7 different methods, *viz*. t-score, F-score, MRMR, SU, PCF, SRC and SVM-RFE, performed equally well in terms of statistical significance of the GSAQ enrichment testing using Inverse normal method (Fig. 3a1). For other methods like RF and GR, the gene sets of sizes 100–300 are more statistically enriched through GSAQ approach as compared to larger gene sets. However, all gene sets selected by IG are not enriched with the underlying salinity QTLs (Fig. 3a1). It can be noted that simple univariate gene selection methods, *i.e*. t-score and F-score, are equally competitive with multivariate (MRMR) and machine learning approaches like SVM-RFE and RF for providing salinity trait enriched gene sets (Fig. 3a1).

Further, the −log10 (*p-values*) from GSAQ approach for SRC is found to be greater than that of other methods followed by t-score, F-score, MRMR and SVM-RFE. This indicate that gene sets selected by SRC are much more enriched with background salinity QTLs. The superiority of SRC in terms of performance may be expected due to its non-parametric nature. Further for SRC, the −log10 (*p-value*) for the gene set of size 1200 is quite higher than that of other gene sets (Fig. 3a1), which indicate the maximal enrichment of the same gene set with QTLs. Similar interpretations can be made for other abiotic and biotic stresses (Figs 3 and 4). Similar interpretations can be made for other methods used in GSAQ approach (Figures S2–S4).

### Chromosome and QTL wise distributions of genes

Along with the trait specific enrichment analysis of gene sets, the proposed GSAQ approach can also be used to get the chromosome and QTL wise distributions of genes in selected gene sets. For instance, chromosomal distributions of genes in the gene set of size 1000 across all the five-different abiotic and biotic stresses are shown in Supplementary Figure S5. For salinity stress, majority of these salinity responsive genes selected by any gene selection method belong to chromosome numbers 2, 3, 4, 5 and 12 (Supplementary Figure S5). Similar interpretations can also be made for cold, drought, fungal and insect stresses.

The QTL wise distributions of genes in the gene set of size 1000 for all the abiotic and biotic stresses are shown in Supplementary Figures S6 and S7 respectively. Further, the proposed GSAQ approach was able to identify and prioritize QTL candidate genes (*i.e*. genes those have QTL hits) from the selected gene set. In case of salinity stress, most of the QTL candidate genes selected by t-score belong to 8 different QTLs out of 13 unique QTLs (Supplementary Figure S6). For other gene selection methods, majority of QTL candidate genes overlapped within 7 salinity responsive QTLs. Further, it has been found that, the QTL with id AQEM001 has largest number of salinity QTL candidate genes followed by AQEM007 and AQEM009 and this trend is true irrespective of gene selection method (Supplementary Figure S6). Similar interpretations can be made for cold, drought, fungal and insect stresses (Supplementary Figures S6 and S7).

## Discussion

Traditional strategies for single gene analysis involves expression analysis of single gene which is mainly focused on identifying individual genes that exhibit differences between two contrasting traits of interest (*e.g*. stress *vs*. control). Although, they are useful, but fail to consider the underlying trait (QTL) specific enrichment of the genes, that are distributed across an entire network of genes in the selected gene set^7^. The existing GSA approaches mostly focused on, whether the selected gene sets are over represented by differentially expressed genes or known pathways or GO terms through over representation analysis^13,16–18,38^. However, in plant biology, QTLs are considered as a great source of information for conducting an effective breeding experiment, as most of the traits are quantitative in nature and controlled by polygenes. Therefore, we proposed GSAQ approach as an innovative and noble way to conduct enrichment analysis of gene sets with trait specific QTLs.

The proposed GSAQ approach is a new way to perform the enrichment analysis of gene sets to establish genotype (polygenes)-phenotype (quantitative trait) association testing with the help of genetically rich trait specific loci data. Further, it is more biologically appealing to establish association of genes (genotype) in the selected gene set with underlying QTLs (traits/phenotypes). However, in the existing GSVQ approach, the genes are taken as input to the Hypergeometric distribution for performing trait enrichment analysis^5^. This approach violates the basic assumptions (*i.e*. sampling units must be drawn without replacement) of this distribution and expected to have poor performance in terms of gene set enrichment. Further, it also fails to state the underlying null hypothesis on which the test is based on. Hence, the proposed GSAQ approach is found to be more successful and effective to detect trait specific QTLs enriched gene sets as it is based on statistically strong null hypothesis.

Further, the proposed GSAQ approach is based on testing a competitive null hypothesis using resampling procedure for possible rejection of competitive *H*_0_. In this approach, *H*_0_ was tested against *H*_1_ with the help of 2 × 2 table method and gene sampling model. This allows one to statistically test the selected gene set for enrichment with the underlying QTLs (*i.e*. rejection of null hypothesis of random association of selected genes with QTLs). Further, a *p-value* was computed for a selected gene set, which is more scientific and statistically meaningful to genome researchers and experimental biologists (as value lies between 0 and 1). The gene sets with lower *p-values* are considered as more enriched with the underlying trait specific QTLs and *vice-versa*. The comparative analysis has shown that the proposed GSAQ approach performs better than existing GSVQ technique for trait specific gene sets enrichment testing. Further, GSAQ approach is more statistically sound, as it satisfied the underlying assumptions of Hypergeometric distribution and 2 × 2 contingency tables. Moreover, the developed GSAQ R package is also flexible in detecting QTL enriched gene sets, as four statistically strong options are available to obtain the *p-values* for selected gene sets.

We also demonstrated the performance of the proposed GSAQ approach for performing QTL enrichment test for the selected gene sets on real crop data sets subjected to various complex abiotic and biotic stresses. There are both challenges and advantages in analyzing these crop datasets. For crops, there are typically limited experimental data available and relatively little literature is available for guidance^5^. The application of GSAQ on complex abiotic and biotic stress scenarios indicated that, it consistently and successfully detects the QTLs enriched gene sets as compared to the existing approach, when the background QTL data is well defined and sufficiently available.

It may be noted that the proposed GSAQ technique is a two stage approach. First, it deals with the selection of gene sets from large gene space by using gene selection methods. Second, it assesses the QTL enrichment significance of gene sets by using the resampling procedure under a gene sampling model and thus provides a suitable statistical framework for testing competitive null hypothesis.

Further, the proposed GSAQ approach has number of advantages when compared with single gene-QTL analysis. First, it eases the interpretation of a large scale experiment by identifying trait specific enriched gene sets. In this, rather than focusing on individual QTL hit genes, researchers can focus on gene sets (polygenes), which tend to be more reproducible and more interpretable (for quantitative traits). Further, the multiple testing of hypothesis problem is well tackled in the proposed approach, as it takes gene set as a functional unit for enrichment analysis. Second, GSAQ is statistically sound as it is based on a competitive null hypothesis and gene sampling model. Further, it considers the genes present in both selected as well as not selected gene sets, while performing trait specific enrichment analysis.

Third, GSAQ approach helps in prioritizing QTL candidate genes or QTL enriched gene sets under a sound computational setup, which would be very helpful in unraveling genotype-to-phenotype relationships. Gene set enrichment testing is well developed in human genetics, where known biological pathways or ontology are taken into account. However, in plant biology and breeding, QTL candidate genes or trait specific enriched gene sets identified through this proposed GSAQ technique will be more effective for developing specific trait or stress tolerant crop cultivars. Fourth, the *NQhits* statistic and statistical significance values computed through the GSAQ approach may be considered as biologically relevant criteria for performance analysis of gene selection methods. However, subject classification accuracy or error rate was a widely used criterion for performance evaluation of gene selection methods^1,19–26^. This may be a statistically necessary but may not be biologically sufficient criterion. Therefore, the proposed GSAQ approach provided two excellent biological relevant criteria for performance evaluation of gene selection methods under a strong statistical framework.

In conclusion, the proposed gene set analysis with trait specific QTLs can be considered as a valuable tool for performing gene(s) enrichment analysis in plant breeding context. Further, the GSAQ approach provides a valuable platform for integrating the GE data with genetically rich QTL data to identify potential QTL enriched gene sets or set of QTL candidate genes, which may act as valuable input or hypothesis for the plant breeders for designing breeding experiments.

In this article, we have statistically established the credibility of the proposed method (GSAQ) by comparing its performance with the only existing approach (GSVQ) through a statistically strong criterion, *i.e*. FDR, in five different stress scenarios in rice. But, in case of crop biotechnology and breeding, very little amount of work has been done to confirm these results. However, these results can provide guidelines to the biotechnologists and breeders to validate the *in silico* results in a wet lab conditions. Moreover, the proposed GSAQ approach can also be used for other expression data analysis like RNA-seq data analysis, if the reference genome is well annotated.

### Data availability

All secondary datasets used in this study are publicly available.

## Electronic supplementary material

Supplementary Information

Table S2

Table S5
