# Supplementary material for: Statistical Approach for Gene Set Analysis with Trait Specific Quantitative Trait Loci
Source: Sci Rep. 2018 Feb 5;8:2391. doi: 10.1038/s41598-018-19736-w (PMC5799309; doi:10.1038/s41598-018-19736-w)
Supplement: Supplementary file 1 — Supplementary Information [file 41598_2018_19736_MOESM1_ESM.doc]

Supplementary information of **“Statistical Approach for Gene Set Analysis with Trait Specific Quantitative Trait Loci”**

Samarendra Das

Anil Rai

D C Mishra

Shesh N Rai^*^

**Document S1: Meta-analysis of** **Gene Expression datasets**

The Gene Expression (GE) samples related to a set of three abiotic stresses (salinity, cold and drought) and two biotic stresses (fungal and insect) were obtained from GE Omnibus database of NCBI ([http://www.ncbi.nlm.nih.gov](http://www.ncbi.nlm.nih.gov/geo)) with platform GPL2025. This platform contains as much as 191 microarray experiments (series) comprising 3096 samples/subjects of rice (Oryza sativa L.) as compared to other platforms*.* Among these samples/subjects, 359 experimental samples related to different biotic and abiotic stresses for rice were collected. Further, to remove outlier samples, meta-analysis was performed individually for each of the stresses.

In meta-analysis, the GE samples with mean (*µ*) ≥ *µ_0_* and standard deviation (*σ*) ≤ *σ_0_* were retained for further study and other samples, which do not satisfy this condition were considered as outliers. The values of *µ_0_* and *σ_0_* were chosen in such a way that the uniformity in color of the correlation plot (among samples) is observed at these parameters setting. For salinity stress, the microarray samples with *µ* ≥ 5.23 and *σ* ≤ 2.53 were selected, as uniformity of colors in the correlation plot for this stress is observed for these parameters setting (Figure S1A). Similarly, for cold stress, the micro-array samples with *µ* ≥ 5.28 and *σ* ≤ 2.52 were selected. For drought stress, the micro-array samples with *µ* ≥ 5.34 and *σ* ≤ 2.51 were selected. For fungal stress, the micro-array samples with *µ* ≥ 5.58 and *σ* ≤ 2.42 were selected. For insect stress, the microarray samples with *µ* ≥ 5.51 and *σ* ≤ 2.32 were retained for further analysis. At these parameters settings, the homogeneity of correlation plot is observed for each of the stresses (Figure S1). In other words, the selected GE samples are observed to be highly homogeneous at these parameters setting, though they were generated over varying experimental conditions. Through the above procedure, 304 GE samples were retained for further study for all the stresses. The detail descriptions about the selected samples is given in Table S2.

**
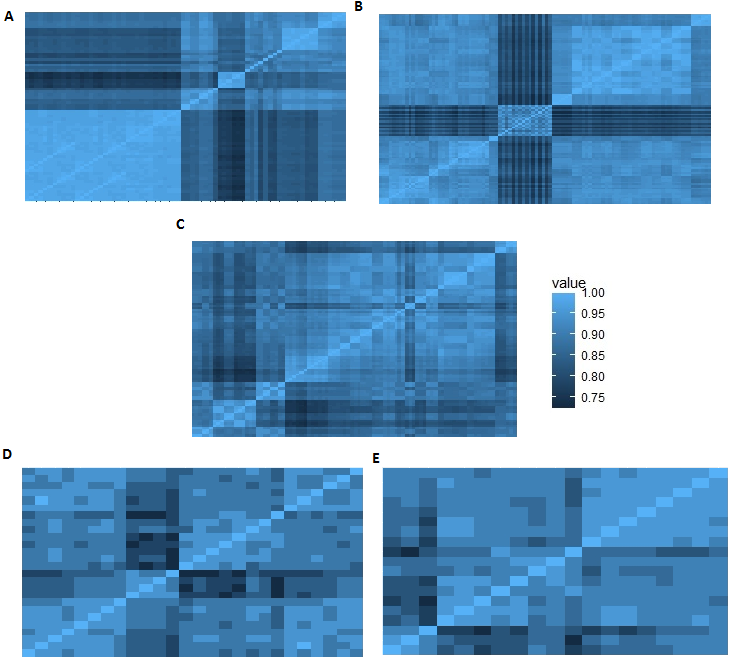
Fig. S1**. Correlation plots for selected microarray samples are shown for (A) salinity, (B) cold, (C) drought, (D) fungal and (E) insect stresses.

**Document S2: Gene sets selection from high dimensional gene expression data.**

***Preliminary gene selection***

Initially, the GE samples consist expression values of 57,162 genes in rice (for the platform GPL 2025). Further, it is computationally expensive to directly apply the gene selection methods on this huge data with large number of features (Liang *et al*., 2011). Hence, we first employed Fold Change (FC) criteria to filter out unlikely genes involved in five different stresses in rice. In our preliminary selection, we assigned a value of 1 as the |FC| threshold for all the stresses because we did not want to lose some important genes at the preliminary stage. As the expression values of genes are log2 transformed, so, the FC can be computed through (Das *et al*., 2017), FC= *log_2_*
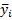
 - *log_2_*
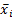
 where,
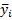
 and
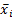
 are the mean expression values of *i-th* gene under stress and control conditions respectively. Through this, we ably reduced the dimensions GE datasets up to few thousands. To further reduce the dimension of the data, we employed t-test. The redundant genes were eliminated from the data by fixing *p-value* cut-off as 0.05 at the preliminary stage. By using both FC and t-test criteria, various genes (as shown in Table S1) were selected for salinity, cold, drought, fungal and insect stresses out of the initial 57,162 genes. Further, GE data on these selected genes (at the preliminary stage) were further used for final gene set selection using different gene set selection methods.

***Gene sets selection using different gene selection methods***

For selection of gene sets, 8 statistical methods, *viz.* t-score, F-score, Maximum Relevance and Minimum Redundancy (MRMR), Information Gain (IG), Gain Ratio (GR), Symmetrical Uncertainty (SU), Pearson’s Correlation Filter (PCF), Spearman’s Rank Correlation (SRC) and two machine-learning methods, *viz*. Random Forest (RF) and Support Vector Machine-Recursive Feature Elimination (SVM-RFE) were used. These 10 gene methods are listed in Table S3 for selection of relevant gene sets. Further, these gene selection methods were applied on the high dimensional GE datasets related to five different stresses in rice.

For t-score, F-score and MRMR methods, we executed the function *GeneSelect* implemented in the developed package GSAQ. For other gene set selection methods, *i.e.* IG, GR, SU, PCF and SRC, we used the functions *information.gain*, *gain.ratio*, *symmetrical.uncertainty*, *linear.correlation* and *rank.correlation* respectively implemented in FSelector pckage of R (Cheng *et al*., 2012). Through these methods, the genes were arranged based on the descending order of their respective scores or weights (gene list). Then different gene sets of sizes, *viz*. 100, 200, 300, …, 2000 were selected from the gene list.

SVM-RFE method was used for ranking of genes from GE data for identification of cancer responsible genes (Guoyon and Elisseeff, 2003). In this algorithm, genes were individually eliminated based on their least significance in classification during SVM training. The objective function, *J* for this classification problem is defined as: $J=\left\|w\right\|^{2}/2$

where, $w$ is kernel width computed by SVM. The Optimal Brain Damage algorithm (Guyon *et al*., 2002) was used to approximate the change in *J*, after deletion of *i-th* gene from the dataset. Further, expanding *J* (up to second order) with the help of Taylor series approximation, the value of *J* was given by

$$\Delta J(i)=(1/2)\frac{\partial^{2}J}{\partial w_{i}^{2}}(\Delta w_{i})^{2}$$

where, $\Delta w_{i}$ is change in weight due to removing *i-th* gene from existing dataset and$\Delta J(i)$is used as weight pruning criterion. Here, the cost function *J* is a quadratic function of $w_{i}$ and both are directly proportional to each other. Hence, measurement of either $w_{i}$ or *J* provides equivalent information. Keeping this in view, $w_{i}^{2}$is used as the ranking criterion for evaluating impact of *i-th* gene on classification. In this process, genes are eliminated with the smallest$w_{i}^{2}$iteratively in a backward elimination manner and whole ranked gene list is prepared at the end. From this gene list, gene sets of sizes, *viz.* 100, 200, 300, …, 2000 are selected to study their QTL enrichment.

For Random Forest (RF), we used the Out-Of-Bag (OOB) error rate for selection of gene sets of varied sizes, *viz.* 100, 200, 300, …, 2000. For this purpose, *varSelRF* function implemented in *VarSelRF* R package (Diaz-Uriarte, 2007) under the following parameters setting, *i.e.* ntree = 500, ntreeIterat = 300, vars.drop.frac = 0.2 was used .

**Table S1.** Summary of datasets used in this study.

| **Sl. No** | **Descriptions** | **#Series** | **#Genes** | **#Sample** | **#Class** | **#QTL** | **# UQTL** | **Stress type** |
| --- | --- | --- | --- | --- | --- | --- | --- | --- |
| A | Salinity Stress | 6 | 6637 | 70 | 2 | 17 | 13 | Abiotic |
| B | Cold Stress | 5 | 8840 | 100 | 2 | 37 | 21 | Abiotic |
| C | Drought Stress | 5 | 9078 | 90 | 2 | 77 | 20 | Abiotic |
| D | Blast Stress | 2 | 7071 | 26 | 2 | 183 | 77 | Biotic |
| E | Brown Plant Hopper | 1 | 7240 | 18 | 2 | 93 | 57 | Biotic |

#Series: Number of GEO series for each dataset; #Genes: Number of genes; #Sample: Number of GEO samples; #S: Number of GE samples belonging to 2 classes (control vs. stress); #QTL: Number of QTLs found for each stress; #UQTL: Number of unique QTLs found in rice for each stress; Stress type: type of stress.

**Table S2**. Gene expression studies and samples used in this study (xlxs).

**Table S3**. List of methods used for gene set selection.

| **Symbol** | **Methods** | **R packages** | **References** |
| --- | --- | --- | --- |
| t | t-score | stat, GSAQ | Cui and Churchil, 2003 |
| F | F-score | MASS, GSAQ |  |
| MRMR | Maximum Relevance Minimum Redundancy | GSAQ | Peng *et al*., 2005 |
| IG | Information Gain | FSelector | Cheng *et al*., 2012 |
| GR | Gain Ratio | FSelector | Cheng *et al*., 2012 |
| SU | Symmetrical Uncertainty | FSelector | Cheng *et al*., 2012 |
| PCF | Linear Correlation Filter | FSelector | Cheng *et al*., 2012 |
| SRC | Spearman’s Rank Correlation | FSelector | Cheng *et al*., 2012 |
| RF | Random Forest | VarSelRF | Diaz-Uriarte, 2007 |
| SVM | SVM-RFE | e1071 | Guyon *et al*., 2002; Liang *et al*., 2011 |

Symbol: Abbreviation used for each method; t: t-test; F: F-score; MRMR: Maximum Relevance Minimum Redundancy; IG: Information gain; GR: Gain ratio; SU: Symmetrical Uncertainty; PCF: Pearson’s Correlation Filter; SRC: Spearman’s Rank Correlation filter; RF: Random Forest; SVM: Support Vector Machine with recursive Feature Elimination; GSAQ is the developed R package.

**References**

Liang Y, Zhang F, Wang J, Joshi T, Wang Y, Xu D (2011) Prediction of Drought-Resistant Genes in Arabidopsis thaliana Using SVM-RFE. PLoS ONE **6**(**7**): e21750; 10.1371/journal.pone.0021750.

Cui,X. & Churchill,G. (2003) Statistical tests for differential expression in cDNA microarray experiments. *Genome Biol*., **4**(**4**), 210.

Peng,H. *et al*. (2005) Feature selection based on mutual information: Criteria of max-dependency, max-relevance and min-redundancy. *IEEE Trans Pattern Anal. Mach. Intell.*, **27**(**8**), 1226–38.

Cheng,T. *et al*. (2012) FSelector: a Ruby gem for feature selection. *Bioinformatics*, **28**(**21**), 2851–2852.

Guyon,I. *et al*. (2002) Gene selection for cancer classification using support vector machines. *Machine Learning*, **46**, 389–422.

Guoyon,I. & Elisseeff,A. (2003) An introduction to variable and feature selection. *J Mach Learn Res*., **3**, 1157-1182.

Diaz-Uriarte,R. (2007). GeneSrF and varSelRF: a web-based tool and R package for gene selection and classification using random forest. *BMC Bioinformatics*, **8**, 328.

Das,S., Meher,P.K., Pradhan,U.K. & Paul,A.K. (2017) Inferring gene regulatory networks using Kendall’s tau correlation coefficient and identification of salinity stress responsive genes in rice. *Curr. Sci.*, **112**(**6**), 1257-62.

**Table S4.** Number of gene samples and sizes of gene sample for each selected gene set for GSAQ analysis.

| **Sl. No.** | **Selected gene set size (*n*)** | **Gene sample size (*m)*** | **No. of gene samples (*K*)** |
| --- | --- | --- | --- |
| 1 | 100 | 85 | 50 |
| 2 | 200 | 170 | 50 |
| 3 | 300 | 255 | 50 |
| 4 | 400 | 340 | 60 |
| 5 | 500 | 425 | 60 |
| 6 | 600 | 510 | 60 |
| 7 | 700 | 595 | 60 |
| 8 | 800 | 680 | 70 |
| 9 | 900 | 765 | 70 |
| 10 | 1000 | 850 | 70 |
| 11 | 1100 | 935 | 70 |
| 12 | 1200 | 1020 | 80 |
| 13 | 1300 | 1105 | 80 |
| 14 | 1400 | 1190 | 80 |
| 15 | 1500 | 1275 | 90 |
| 16 | 1600 | 1360 | 90 |
| 17 | 1700 | 1445 | 90 |
| 18 | 1800 | 1530 | 100 |
| 19 | 1900 | 1615 | 100 |
| 20 | 2000 | 1700 | 100 |

**Table S5**. False discovery rate computed for each gene set through GSVQ and GSAQ approaches for all stresses in rice (*Oryza sativa* L.). (xlxs)

**Document S3: Stress/trait specific Quantitative Trait Loci information for rice (*Oryza sativa* L.)**

The list of trait specific unique Quantitative Trait Loci (QTL) for the abiotic stresses *viz*. salinity, drought and cold and biotic stresses *viz.* fungal (blast) and insect (brown plant hopper) for rice were collected from the Gramene QTL database (<http://www.gramene.org/qtl/>) (Ni *et al*., 2009). Then, the genomic regions of these QTLs (for each stress) were mapped to rice genome using Gramene annotation of rice genome of MSU Rice Genome Annotation (Osa1) Release 6 (Ouyang *et al*. 2007). Here, we incorporated the QTL information with transcription profiles to perform GSAQ analysis to various stresses. For instance, a given QTL, there may be 25–30 genes per cM (∼270 kbp in rice) (Khurana and Gaikwad, 2005). The unique QTLs (non-overlapping) are used for performing GSAQ analysis of the selected gene sets for these stresses. The lists of the unique QTLs for each of the four stresses are given in Table S6-S10.

**Table S6**. List of unique salinity responsive QTLs in rice (*Oryza sativa L.*)

| **Sl. No.** | **QTL ID** | **Chr. No.** | **Start** | **End** | **Note** |
| --- | --- | --- | --- | --- | --- |
| 1 | AQEM001 | 1 | 33956950 | 37713775 |  |
| 2 | AQEM006 | 1 | 9820009 | 11232822 |  |
| 3 | AQGR001 | 1 | 38530957 | 38531467 |  |
| 4 | AQGR002 | 3 | 22798284 | 22830744 |  |
| 5 | AQCL001 | 3 | 484860 | 485333 |  |
| 6 | AQEM009 | 4 | 19928370 | 22355854 |  |
| 7 | AQCL002 | 4 | 33663984 | 33664487 |  |
| 8 | AQCL003 | 5 | 18874932 | 18875558 |  |
| 9 | AQCL004 | 6 | 22862400 | 22862821 |  |
| 10 | AQEM002 | 6 | 21605889 | 24919236 |  |
| 11 | AQEM003 | 7 | 4573316 | 7739951 |  |
| 12 | AQEM004 | 7 | 2633784 | 4575215 |  |
| 13 | AQEM007 | 9 | 14362062 | 17837010 |  |

Sl. No.: Serial number of the unique QTL; QTL ID: Published qtl id; Chr. No.: Chromosome number of the QTL; Start: start position of the QTL in terms of base pairs (bp); End: end position of the QTL in terms of length of bps.

**Table S7**. List of cold responsive unique QTLs for rice (*Oryza sativa L.*)

| Sl. No. | QTL ID | Chr. | Start | End |
| --- | --- | --- | --- | --- |
| 1 | CQAA8 | 4 | 688353 | 6574518 |
| 2 | AQDU004 | 11 | 932068 | 932221 |
| 3 | CQP8 | 11 | 1491600 | 2523808 |
| 4 | AQDU009 | 6 | 5425408 | 5425631 |
| 5 | AQAV003 | 1 | 5558576 | 7445919 |
| 6 | AQDU014 | 12 | 8826555 | 8826855 |
| 7 | AQAV004 | 2 | 11389704 | 12216613 |
| 8 | AQAV002 | 9 | 17719660 | 18810331 |
| 9 | AQF129 | 8 | 19051713 | 22886866 |
| 10 | AQDU002 | 6 | 19499320 | 27252383 |
| 11 | AQDU003 | 8 | 20650060 | 21142502 |
| 12 | CQP7 | 7 | 22857717 | 22885543 |
| 13 | AQDU013 | 11 | 25153466 | 25153681 |
| 14 | AQDU015 | 4 | 26857374 | 29061127 |
| 15 | AQBO001 | 7 | 27159051 | 27159261 |
| 16 | AQAV006 | 5 | 27342022 | 27342124 |
| 17 | CQO3 | 4 | 29155838 | 30445683 |
| 18 | AQDU001 | 4 | 30772388 | 32650528 |
| 19 | CQO1 | 4 | 31276528 | 32772351 |
| 20 | CQAA6 | 1 | 32099566 | 33677892 |
| 21 | CQP1 | 1 | 34651088 | 39949610 |

Sl. No.: Serial number of the QTL; QTL ID: Published qtl id; Chr. No.: Chromosome number of the QTL; Start: start position of the QTL in terms of base pairs (bp); End: end position of the QTL in terms of length of bps.

**Table S8**. List of drought responsive QTLs in rice (*Oryza sativa L.*)

| **Sl. No.** | **QTL ID** | **Chr.** | **Start** | **End** |
| --- | --- | --- | --- | --- |
| 1 | CQAI48 | 4 | 13634515 | 13635012 |
| 2 | AQA046 | 12 | 26017140 | 27489485 |
| 3 | AQHP062 | 8 | 21645663 | 21647445 |
| 4 | AQHP058 | 2 | 10503368 | 10503846 |
| 5 | AQHP059 | 4 | 31662839 | 31663326 |
| 6 | AQHP082 | 6 | 6718648 | 9537772 |
| 7 | AQHP083 | 7 | 13074864 | 13075056 |
| 8 | AQHP065 | 1 | 29184260 | 29184844 |
| 9 | AQHP081 | 4 | 8610617 | 8611256 |
| 10 | AQHP079 | 3 | 15469002 | 19412007 |
| 11 | AQAN005 | 8 | 20094533 | 20094695 |
| 12 | AQHP066 | 2 | 29761981 | 29762453 |
| 13 | AQHP069 | 3 | 22798284 | 35828040 |
| 14 | AQHP068 | 2 | 10503368 | 19866086 |
| 15 | AQAN001 | 5 | 27342022 | 28610866 |
| 16 | AQHP078 | 11 | 4413928 | 4415836 |
| 17 | AQHP067 | 2 | 27034342 | 27035328 |
| 18 | AQHP085 | 9 | 20481606 | 20482133 |
| 19 | AQHP061 | 6 | 2560318 | 2561213 |
| 20 | AQHP087 | 11 | 19565059 | 19565672 |

Sl. No.: Serial number of the QTL; QTL ID: Published qtl id; Chr.: Chromosome number of the QTL; Start: start position of the QTL in terms of base pairs (bp); End: end position of the QTL in terms of length of bps.

**Table S9.** List of blast (fungal) responsive unique QTLs in rice (*Oryza sativa L.*).

| **Sl. No.** | **QTL ID** | **Chr.** | **Start** | **Stop** |
| --- | --- | --- | --- | --- |
| 1 | AQAF003 | 1 | 1,98,822 | 18,91,260 |
| 2 | AQEN002 | 1 | 50,94,276 | 1,10,77,990 |
| 3 | AQAH002 | 1 | 2,41,86,290 | 2,91,84,844 |
| 4 | AQEN079 | 1 | 50,94,276 | 50,95,699 |
| 5 | AQGJ001 | 1 | 2,94,46,995 | 2,94,47,853 |
| 6 | AQAF011 | 1 | 3,10,46,003 | 3,10,47,458 |
| 7 | AQAF013 | 1 | 3,30,53,493 | 4,00,65,325 |
| 8 | AQAF015 | 1 | 3,44,70,620 | 4,00,65,325 |
| 9 | AQAF017 | 1 | 3,44,70,620 | 3,77,13,775 |
| 10 | AQAQ008 | 1 | 3,07,37,705 | 4,05,67,354 |
| 11 | AQAF006 | 1 | 74,45,627 | 7445919 |
| 12 | AQEN001 | 1 | 1,46,20,467 | 3,49,40,769 |
| 13 | AQAF007 | 1 | 79,70,722 | 79,70,839 |
| 14 | AQCT001 | 1 | 47,38,488 | 3,01,70,285 |
| 15 | AQEN011 | 1 | 1,46,20,467 | 2,17,01,719 |
| 16 | AQEN051 | 1 | 2,05,98,332 | 2,05,99,810 |
| 17 | AQCT002 | 2 | 3,56,61,689 | 3,56,62,199 |
| 18 | AQAQ001 | 2 | 3,56,61,689 | 3,56,62,199 |
| 19 | AQAF026 | 2 | 3,46,52,316 | 3,51,36,068 |
| 20 | AQEN069 | 2 | 2,74,82,581 | 3,11,07,173 |
| 21 | AQEN070 | 2 | 2,74,82,581 | 27,483,257 |
| 22 | AQEN039 | 3 | 4,84,860 | 4,85,333 |
| 23 | AQEN012 | 3 | 4,84,860 | 1,450,227 |
| 24 | AQEN003 | 3 | 4,84,860 | 34,96,275 |
| 25 | AQAQ020 | 3 | 2,51,28,239 | 25,128,864 |
| 26 | AQAF029 | 3 | 2,30,88,332 | 2,45,95,466 |
| 27 | AQGJ003 | 4 | 86,10,617 | 86,11,256 |
| 28 | AQEN063 | 4 | 3,16,62,839 | 3,16,63,326 |
| 29 | AQAQ015 | 4 | 86,10,617 | 1,12,34,543 |
| 30 | CQAC1 | 4 | 1,99,28,370 | 2,23,55,854 |
| 31 | AQEN061 | 4 | 2,00,87,103 | 2,00,87,362 |
| 32 | AQAQ024 | 5 | 2,25,79,390 | 2,25,80,355 |
| 33 | AQCT003 | 5 | 20,91,276 | 2,782,394 |
| 34 | AQEN041 | 6 | 23,63,670 | 23,63,704 |
| 35 | AQEN005 | 6 | 23,63,670 | 67,20,901 |
| 36 | AQAQ021 | 6 | 2,90,27,995 | 3,09,45,628 |
| 37 | AQEN014 | 6 | 95,36,259 | 2,44,55,212 |
| 38 | AQGJ023 | 6 | 25,60,318 | 62,84,636 |
| 39 | AQEN059 | 6 | 95,36,259 | 95,37,772 |
| 40 | AQGJ008 | 6 | 2,67,07,816 | 26,708,549 |
| 41 | AQCT004 | 6 | 62,83,401 | 6,928,661 |
| 42 | AQAH001 | 6 | 69,27,624 | 6,928,661 |
| 43 | AQEN044 | 7 | 2,94,66,368 | 2,94,67,498 |
| 44 | AQEN007 | 7 | 1,30,74,864 | 29,467,498 |
| 45 | AQAF030 | 7 | 15,36,133 | 2,317,976 |
| 46 | AQEN033 | 7 | 15,36,133 | 1,537,879 |
| 47 | AQAF033 | 7 | 2,54,72,688 | 2,65,29,185 |
| 48 | AQGJ010 | 7 | 2,67,04,922 | 29,467,498 |
| 49 | AQAQ016 | 7 | 1,75,25,817 | 18,686,761 |
| 50 | AQAF031 | 7 | 23,16,691 | 7,232,998 |
| 51 | AQGJ026 | 7 | 71,24,042 | 7,124,718 |
| 52 | AQAF035 | 8 | 41,05,519 | 53,27,118 |
| 53 | AQEN015 | 8 | 41,05,519 | 1,74,38,003 |
| 54 | AQAF034 | 8 | 41,05,519 | 41,06,001 |
| 55 | AQEN037 | 8 | 41,05,519 | 4,106,001 |
| 56 | AQAF038 | 9 | 1,46,48,372 | 20,174,430 |
| 57 | CQAC3 | 9 | 96,29,362 | 10,801,158 |
| 58 | AQAQ022 | 9 | 12,71,123 | 10,801,158 |
| 59 | AQAF040 | 9 | 1,77,19,660 | 18,810,331 |
| 60 | AQAF041 | 9 | 1,99,46,740 | 20,482,185 |
| 61 | AQGJ027 | 9 | 1,88,10,067 | 1,88,10,331 |
| 62 | AQAF042 | 9 | 2,11,89,110 | 22,196,064 |
| 63 | AQCT006 | 10 | 2,09,76,812 | 20,978,165 |
| 64 | AQEN067 | 11 | 2,03,36,572 | 20,337,612 |
| 65 | AQEN016 | 11 | 1,81,78,768 | 20,337,612 |
| 66 | AQAQ017 | 11 | 1,36,71,613 | 28,412,347 |
| 67 | AQEN081 | 11 | 1,81,78,768 | 18,179,510 |
| 68 | AQAO001 | 11 | 1,78,08,335 | 22,816,523 |
| 69 | AQGJ013 | 11 | 66,86,166 | 6,687,145 |
| 70 | AQCT007 | 11 | 46,24,598 | 46,26,888 |
| 71 | AQAQ009 | 12 | 5,32,909 | 1,595,325 |
| 72 | AQEN017 | 12 | 77,29,365 | 23,775,487 |
| 73 | AQEN047 | 12 | 77,29,365 | 7,729,855 |
| 74 | CQAC4 | 12 | 1,10,58,522 | 18,867,702 |
| 75 | AQCT008 | 12 | 77,29,365 | 13,429,507 |
| 76 | AQAQ011 | 12 | 77,29,365 | 77,29,855 |
| 77 | AQEN072 | 12 | 15,94,823 | 15,95,325 |

Sl. No.: Serial number of the unique QTL; QTL ID: Published qtl id; Chr. No.: Chromosome number of the QTL; Start: start position of the QTL in terms of base pairs (bp); End: end position of the QTL in terms of length of bps.

**Table S10**. List of brown plant hopper (insect) responsive unique QTLs in rice (*Oryza sativa L.*).

| **Sl. No.** | **QTL ID** | **Chr.** | **Start** | **Stop** |
| --- | --- | --- | --- | --- |
| 1 | AQAP053 | 2 | 7,44,663 | 7,45,178 |
| 2 | AQAP027 | 6 | 17,64,586 | 18,22,651 |
| 3 | CQAM3 | 1 | 4,29,53,262 | 42,955,596 |
| 4 | AQAP058 | 10 | 53,52,766 | 1,58,02,326 |
| 5 | AQAP009 | 8 | 1,39,27,893 | 2,28,86,866 |
| 6 | AQBA009 | 6 | 67,18,648 | 67,20,901 |
| 7 | AQBA003 | 8 | 2,78,22,512 | 2,78,25,271 |
| 8 | AQBA005 | 4 | 3,36,63,984 | 3,46,98,383 |
| 9 | AQW015 | 12 | 2,74,88,270 | 2,74,89,485 |
| 10 | AQW012 | 11 | 2,31,54,725 | 2,31,55,291 |
| 11 | AQBA002 | 6 | 67,18,648 | 6,720,901 |
| 12 | AQAP023 | 5 | 1,88,74,932 | 22,580,355 |
| 13 | AQAP040 | 6 | 1,70,54,655 | 17,055,184 |
| 14 | AQAP032 | 10 | 2,10,98,188 | 2,10,99,881 |
| 15 | AQAP054 | 3 | 32,36,247 | 3,236,745 |
| 16 | AQAP048 | 4 | 3,06,30,093 | 3,06,30,917 |
| 17 | AQAP036 | 12 | 2,61,07,904 | 26,992,979 |
| 18 | AQAP050 | 11 | 1,36,71,613 | 2,31,55,291 |
| 19 | AQAP051 | 2 | 2,58,65,334 | 27,610,063 |
| 20 | AQAP043 | 5 | 2,25,79,390 | 29,285,656 |
| 21 | AQAP018 | 8 | 2,28,85,196 | 26,282,308 |
| 22 | AQAP035 | 9 | 2,21,94,746 | 2,21,96,064 |
| 23 | AQAP042 | 9 | 2,21,94,746 | 22,196,064 |
| 24 | AQAP029 | 6 | 39,22,784 | 26,708,549 |
| 25 | AQW010 | 6 | 67,18,648 | 19,338,095 |
| 26 | AQBA020 | 6 | 67,18,648 | 1,93,38,095 |
| 27 | AQAP015 | 1 | 2,76,25,475 | 2,91,84,844 |
| 28 | AQAP028 | 8 | 89,23,052 | 8,924,004 |
| 29 | CQAM2 | 6 | 41,60,454 | 8,066,358 |
| 30 | AQW007 | 3 | 57,29,669 | 7,350,653 |
| 31 | AQAP004 | 3 | 3,03,13,472 | 3,03,15,075 |
| 32 | CQT1 | 4 | 35,46,753 | 14,707,274 |
| 33 | AQAP030 | 11 | 1,36,71,613 | 28,412,347 |
| 34 | AQAP045 | 1 | 3,96,87,395 | 4,05,67,354 |
| 35 | AQAP005 | 5 | 52,55,880 | 52,56,140 |
| 36 | AQBA017 | 1 | 1,46,20,467 | 14,626,881 |
| 37 | AQAP056 | 6 | 31,68,314 | 54,25,631 |
| 38 | AQAP057 | 7 | 1,75,25,817 | 2,57,75,868 |
| 39 | AQAP039 | 1 | 10,39,086 | 10,39,868 |
| 40 | AQBA007 | 2 | 1,98,65,083 | 2,45,66,182 |
| 41 | AQAU001 | 2 | 89,84,645 | 18,249,617 |
| 42 | CQT2 | 3 | 3,19,45,962 | 35,710,936 |
| 43 | AQW013 | 12 | 1,96,28,443 | 19,628,925 |
| 44 | AQAP034 | 11 | 57,06,417 | 57,06,935 |
| 45 | AQAP041 | 2 | 45,24,663 | 5,263,536 |
| 46 | AQAP055 | 4 | 2,01,71,917 | 2,01,73,040 |
| 47 | AQW014 | 12 | 1,96,28,443 | 19,628,925 |
| 48 | AQBA022 | 1 | 1,46,20,467 | 14,626,881 |
| 49 | AQW008 | 4 | 2,41,65,104 | 24,165,408 |
| 50 | AQAU003 | 12 | 5,32,909 | 5,33,313 |
| 51 | AQAP007 | 5 | 52,55,880 | 6,700,408 |
| 52 | AQAU002 | 10 | 2,14,02,080 | 23,031,714 |
| 53 | AQAP031 | 8 | 1,39,27,893 | 2,06,50,257 |
| 54 | AQAP052 | 2 | 52,62,891 | 69,16,662 |
| 55 | AQBA008 | 4 | 3,16,62,839 | 32,449,446 |
| 56 | AQAP046 | 10 | 1,77,94,267 | 1,98,23,295 |
| 57 | AQAP001 | 1 | 97,01,793 | 1,04,91,821 |

Sl. No.: Serial number of the unique QTL; QTL ID: Published qtl id; Chr. No.: Chromosome number of the QTL; Start: start position of the QTL in terms of base pairs (bp); End: end position of the QTL in terms of length of bps.

**References**

Khurana,P. & Gaikwad,K. (2005) The map based sequence of the rice genome. *Nature* **436**, 793–800. doi: 10.1038/nature03895

Ni,J. *et al*. (2009) Gramene QTL database: development, content and applications. *Database* **2009**: bap005.

Ouyang,S. *et al*. (2007) The TIGR Rice Genome Annotation Resource: improvements and new features. *Nucleic Acids Research* **35**: D883–D887. doi: 10.1093/nar/gkl976

**Document S4: Inputs to GSAQ**

1. Gene Expression dataset ***D*** with *N* genes and *M* samples
2. QTL dataset ***Q*** with rows as *K* QTL ids and columns as chromosome number, start and stop positions of the QTL id.
3. Gene space (list) dataset with *N* genes as rows and columns as chromosome number, start and stop positions of each gene.
4. Gene Selection Method to produce Gene Set *G*. Includes a simple univariate t-test, F-score or correlation (Pearson’s or Spearman’s) measures (or other ranking metric) and a phenotype or profile of interest *C*. (See Supplementary Table S3 for details of gene selection methods). We use only one probe per gene to prevent overestimation of the NQhits statistic.
5. Random Gene samples from *G* with Size *m* drawn without replacement
6. A value *S* to decide the number random gene samples drawn from *G*
7. Method for combining individual p-values. Includes five different options for combining the individual p-values obtained from each random gene samples
8. Level of significance value (α) to decide the statistical significance of QTL enrichment of *G*

**
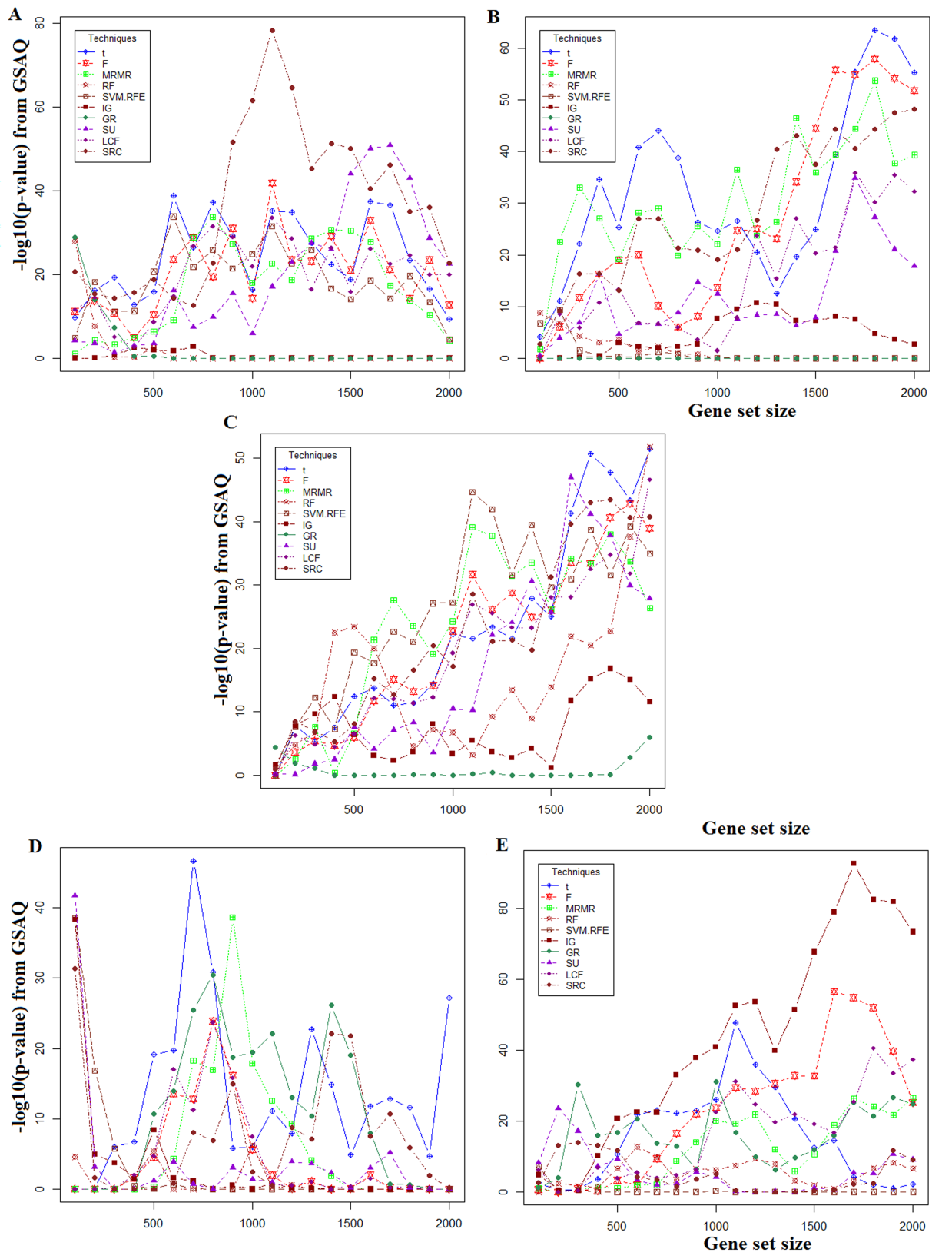
**

**Fig. S2. Distribution of statistical significance values computed from GSAQ approach with Meanp method.** The horizontal axis represents the Gene sets (number genes in the selected gene set) obtained by each of the ten gene selection methods. The vertical axis shows the *negative logarithm of statistical significance values* from the proposed GSAQ approach with Meanp method of combining gene sample *p-values*. Distribution of *p-values* from GSAQ (with Meanp method) are shown for (A) salinity stress, (B) cold stress, (C) drought stress, (D) fungal (Blast) stress and (E) insect stress in rice.

**
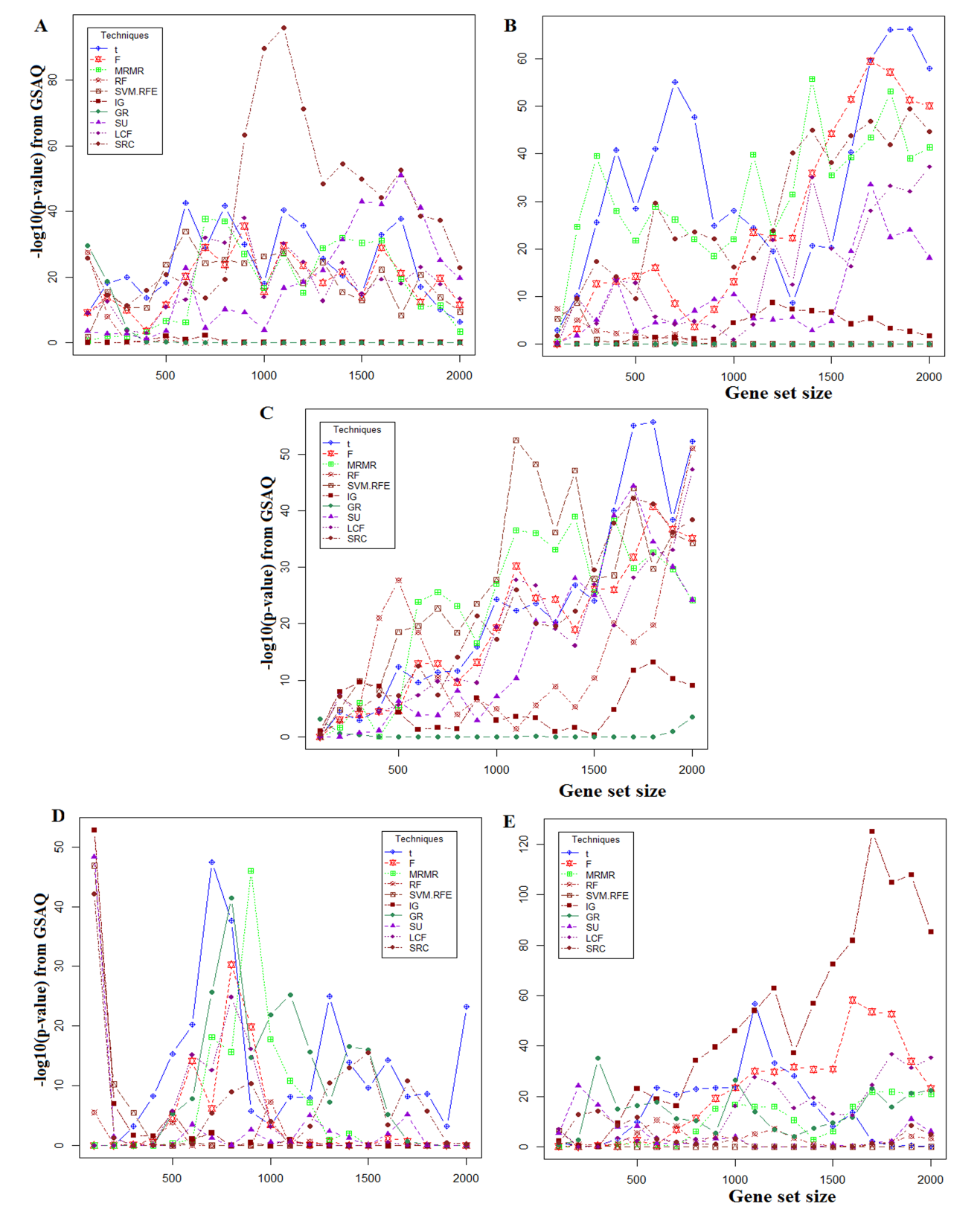
**

**Fig. S3. Distribution of statistical significance values computed from GSAQ approach with Inverse Chi-square method**. The horizontal axis represents the Gene sets (number genes in the selected gene set) obtained by each of the ten gene selection methods. The vertical axis shows the *negative logarithm of statistical significance values* from the proposed GSAQ approach with Inverse normal method of combining gene sample *p-values*. Distribution of *p-values* from GSAQ (with Inverse normal method) are shown for (A) salinity stress, (B) cold stress, (C) drought stress, (D) fungal (Blast) stress and (E) insect (Brown Plant Hopper) stress in rice.

**
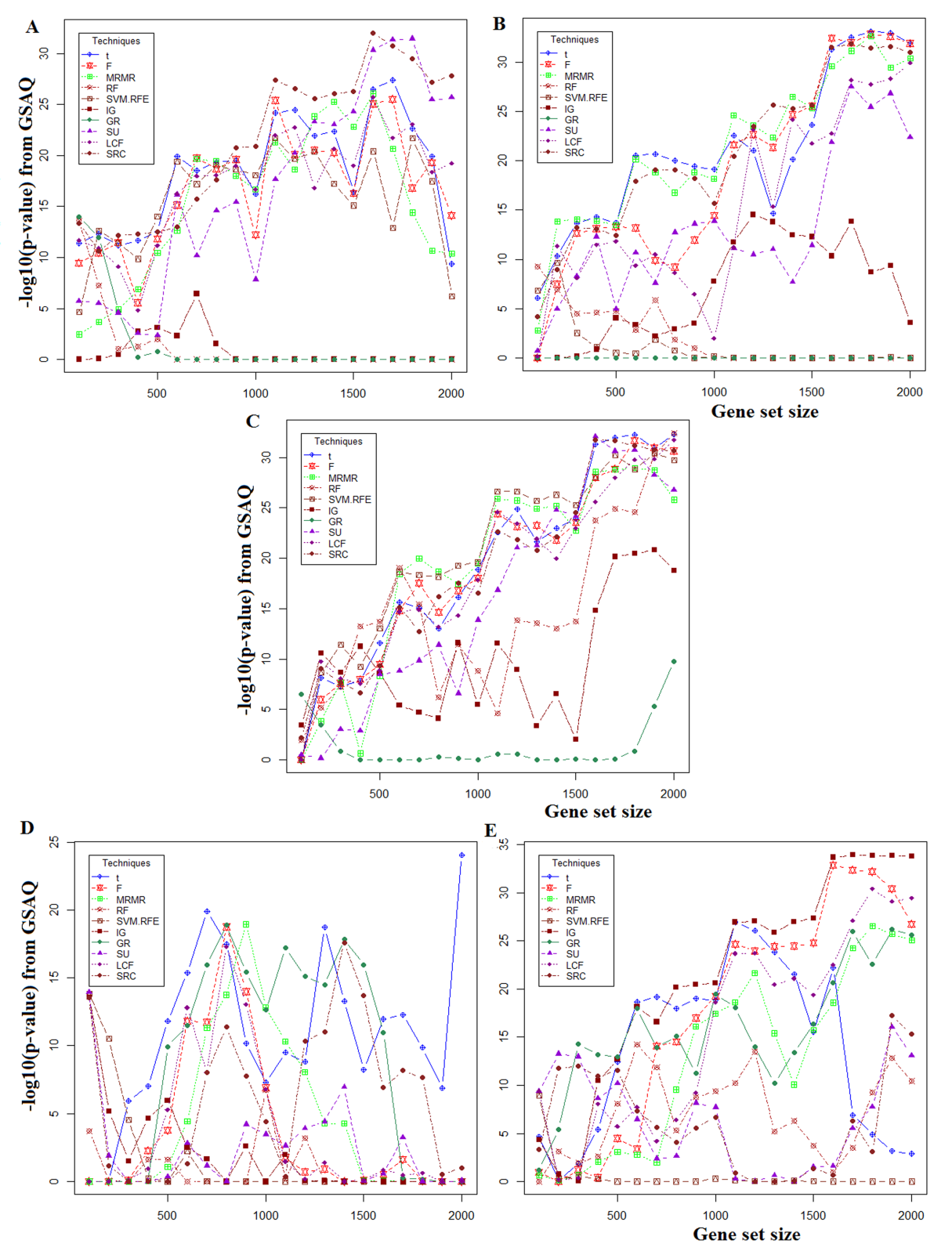
**

**Fig. S4. Distribution of statistical significance values computed from GSAQ approach with Logit method.** The horizontal axis represents the Gene sets (number genes in the selected gene set) obtained by each of the ten gene selection methods. The vertical axis shows the *negative logarithm of statistical significance values* from the proposed GSAQ approach with Logit model method of combining gene sample *p-values*. Distribution of *p-values* from GSAQ (with Logit model) are shown for (A) salinity stress, (B) cold stress, (C) drought stress, (D) fungal stress and (E) insect stress in rice.

**
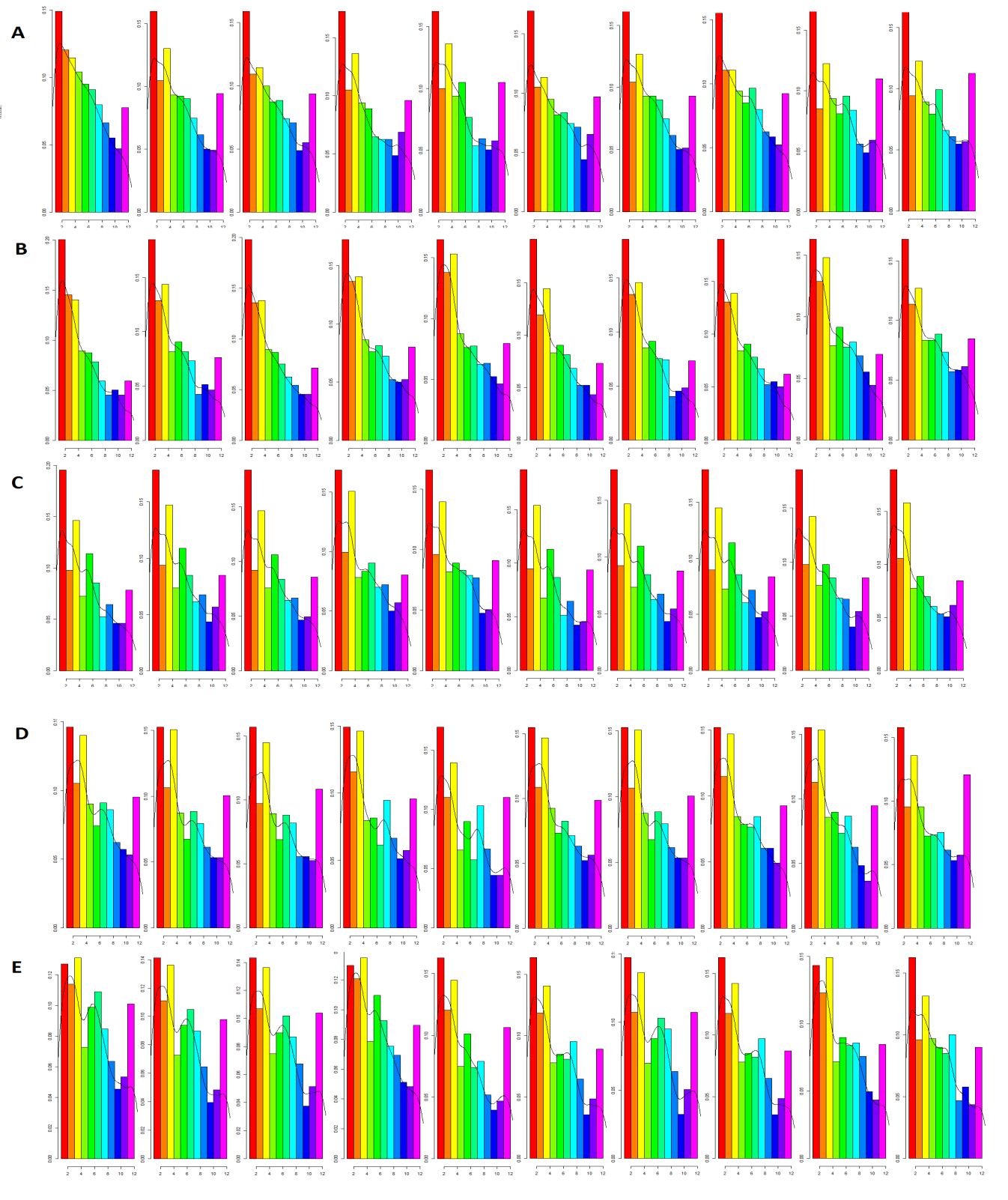
**

**Fig. S5. Chromosomal distribution of genes in the selected gene sets.** The horizontal axis represents the chromosome numbers. The vertical axis shows the *density* of genes in the selected gene set for each chromosome. Each density plot shows the chromosome wise distribution of genes obtained by a gene selection method. Each rows of this figure represents chromosomal distribution of genes in gene set of size 1000 for (A) salinity stress, (B) cold stress, (C) drought stress, (D) fungal stress and (E) insect stress in rice. The plots are arranged by the gene selection methods as given in Table S3.

**
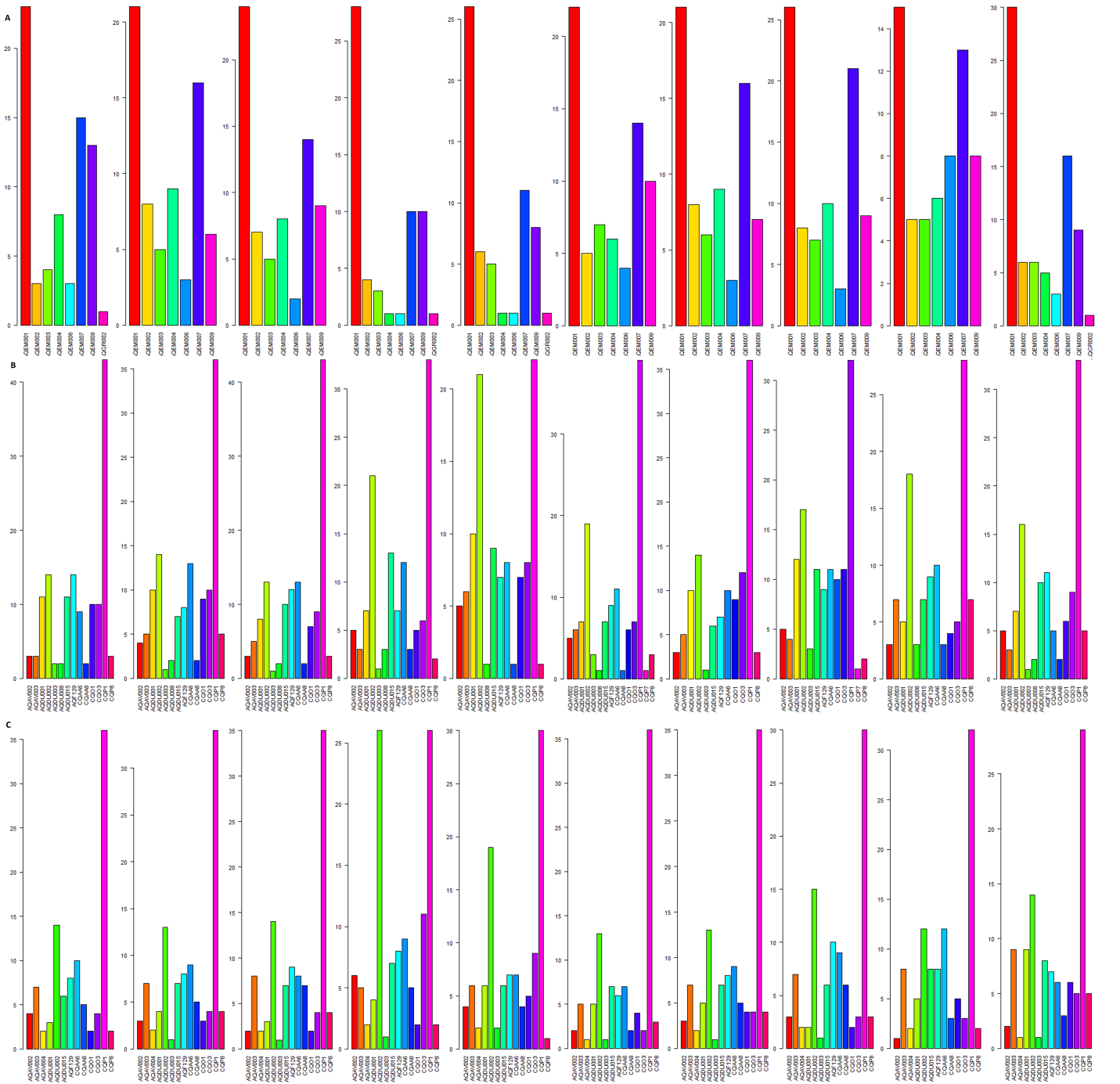
Fig. S6. QTL wise distribution of QTL candidate genes in the selected gene sets for abiotic stresses in rice.** The horizontal axis represents the QTL id. The vertical axis shows the number of genes in the selected gene set for each stress responsive QTLs. Each bar plot shows the QTL wise distribution of QTL candidate genes obtained by a gene selection method. Each rows of this figure represents QTL hit distribution of genes in gene set of size 1000 for respective abiotic stresses (salinity, cold and drought). The bar plots are arranged by the gene selection methods as given in Table S3.

**
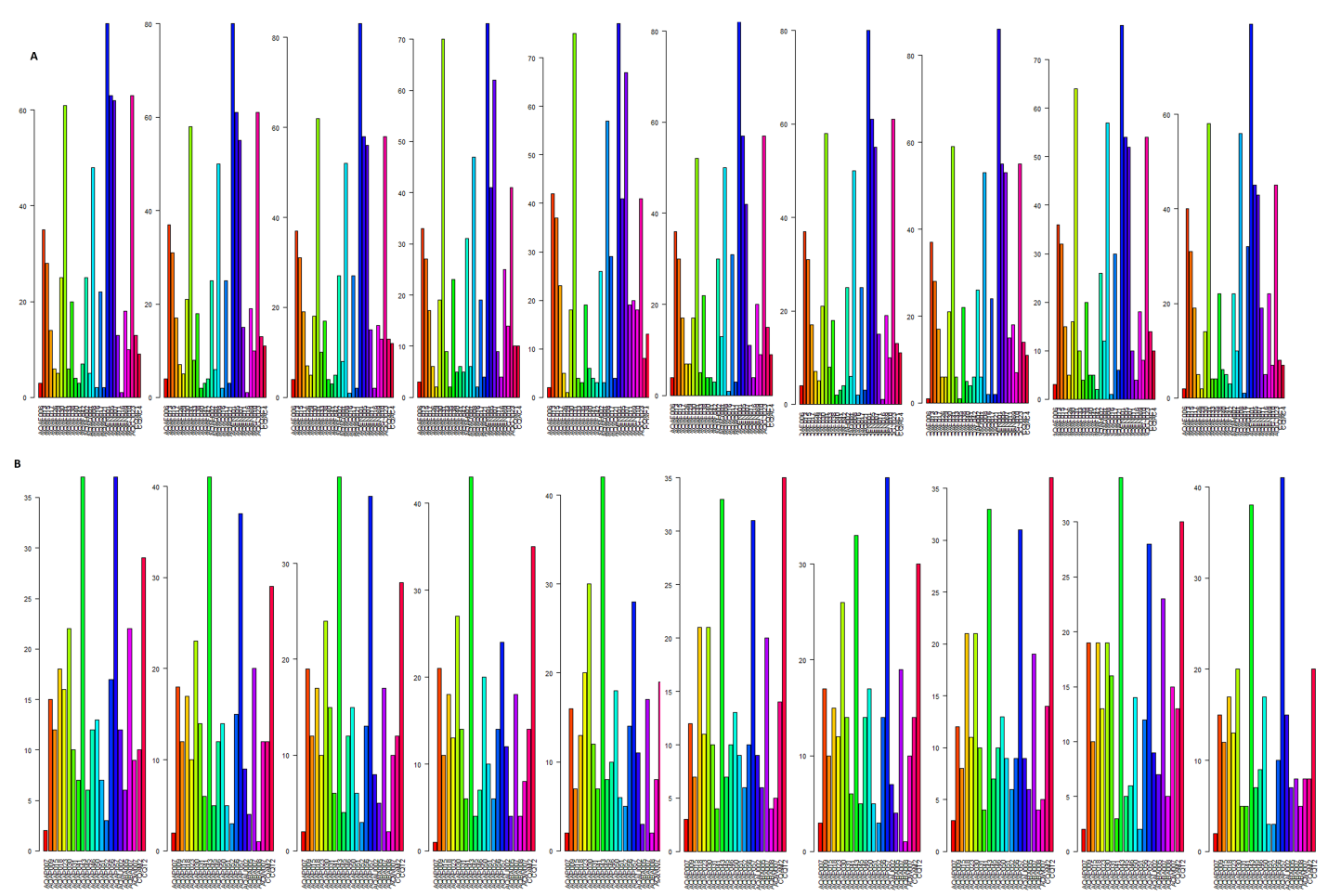
Fig. S7. QTL wise distribution of QTL candidate genes in the selected gene sets for biotic stresses in rice.** The horizontal axis represents the QTL id. The vertical axis shows the number of genes in the selected gene set for each stress responsive QTLs. Each bar plot shows the QTL wise distribution of QTL candidate genes obtained by a gene selection method. Each rows of this figure represents distribution of QTL hit genes in gene set of size 1000 for respective biotic stresses (fungal (A) and insect (B)). The bar plots are arranged by the gene selection methods as given in Table S3.
